# *Discoidin domain receptor* regulates ensheathment, survival and caliber of peripheral axons

**DOI:** 10.1242/dev.200636

**Published:** 2022-12-13

**Authors:** Megan M. Corty, Alexandria L. Hulegaard, Jo Q. Hill, Amy E. Sheehan, Sue A. Aicher, Marc R. Freeman

**Affiliations:** ^1^ Vollum Institute, Oregon Health & Science University, Portland, OR 97239, USA; ^2^ Department of Chemical Physiology & Biochemistry, Oregon Health & Science University, Portland, OR 97239, USA

**Keywords:** Wrapping glia, *Drosophila*, Remak Schwann cell, Multiplexin, Axon ensheathment

## Abstract

Most invertebrate axons and small-caliber axons in mammalian peripheral nerves are unmyelinated but still ensheathed by glia. Here, we use *Drosophila* wrapping glia to study the development and function of non-myelinating axon ensheathment, which is poorly understood. Selective ablation of these glia from peripheral nerves severely impaired larval locomotor behavior. In an *in vivo* RNA interference screen to identify glial genes required for axon ensheathment, we identified the conserved receptor tyrosine kinase Discoidin domain receptor (Ddr). In larval peripheral nerves, loss of Ddr resulted in severely reduced ensheathment of axons and reduced axon caliber, and we found a strong dominant genetic interaction between *Ddr* and the type XV/XVIII collagen *Multiplexin* (*Mp*), suggesting that Ddr functions as a collagen receptor to drive axon wrapping. In adult nerves, loss of Ddr decreased long-term survival of sensory neurons and significantly reduced axon caliber without overtly affecting ensheathment. Our data establish essential roles for non-myelinating glia in nerve development, maintenance and function, and identify Ddr as a key regulator of axon–glia interactions during ensheathment and establishment of axon caliber.

## Teaser

**Highlighted Article:** *In vivo* RNA interference identifies the receptor tyrosine kinase discoidin domain receptor as required for the proper development and function of non-myelinating glia and axonal ensheathment in the fly.

## INTRODUCTION

In complex nervous systems, specialized glial cells ensheathe long axons. Myelination is the most studied type of ensheathment, but unmyelinated axons make up the majority (∼70%) of axons in human peripheral nerves (Ochoa and Mair, 1969; Schmalbruch, 1986). Vertebrate Remak Schwann cells ensheathe and separate unmyelinated axons. These small-caliber axons include autonomic and sensory neuron axons, including nociceptive c-fibers (Griffin and Thompson, 2008). Remak Schwann cells are thought to mediate nerve development and sensory biology, and be important modulators of neurological conditions, including peripheral neuropathies and nerve injuries. However, Remak Schwann cells have remained understudied, in part owing to a lack of selective genetic tools to target this population of cells specifically.

**Fig. 1.**
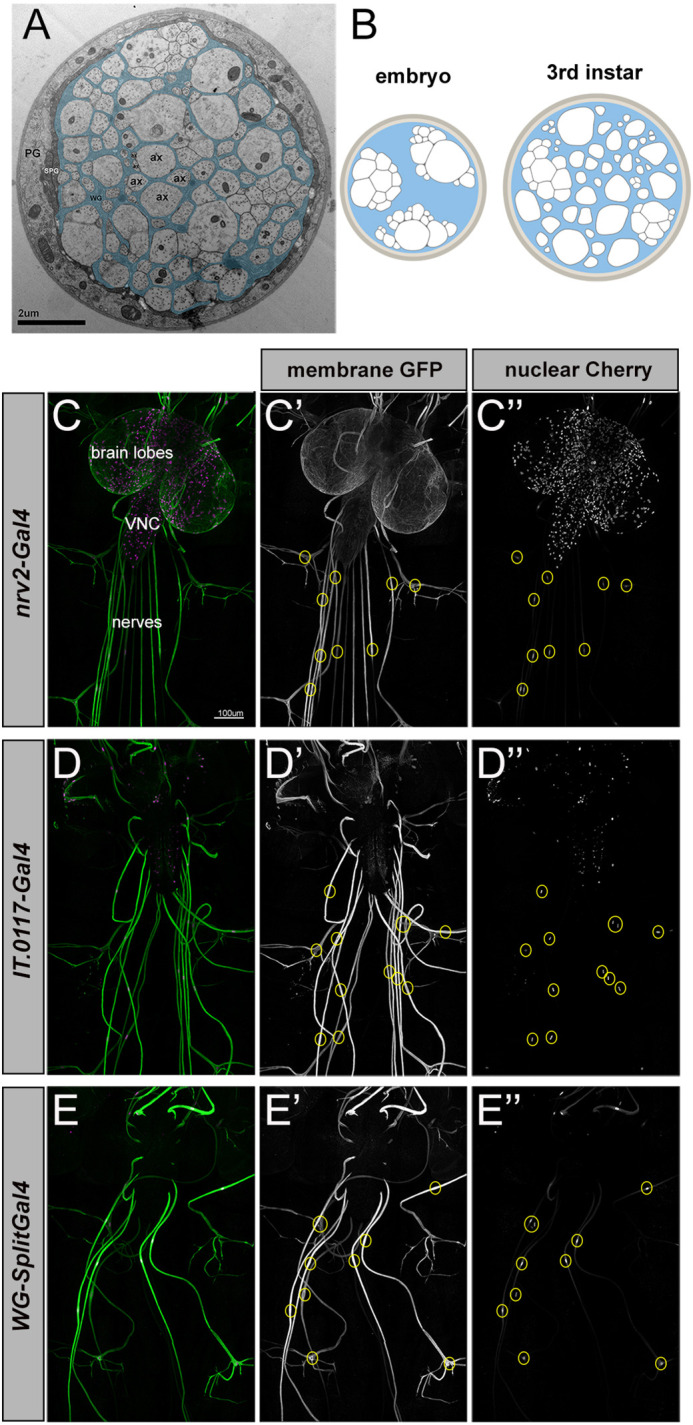
**Construction of *Drosophila* wrapping glia *Split-Gal4* driver.** (A) TEM cross-section of a third instar larval nerve. Light, round profiles of larval axons (ax) are surrounded by darker (pseudo-colored cyan) wrapping glia. Subperineurial (SPG) and perineurial glia (PG; not colored) form the outer layers of the nerve. (B) Schematic of larval nerve cross-sections at embryonic and third instar stages depicting ensheathment status. Axons are depicted in white; wrapping glia coverage is shown in blue. (C-E″) Expression patterns of Gal4 lines in third instar larvae. Each Gal4 depicted is driving a membrane-bound GFP (green) and nuclear mCherry (magenta). Wrapping glia nuclei along nerves are indicated with yellow circles. (C-C″) *Nrv2-Gal4* drives UAS expression exclusively in wrapping glia in the PNS (nuclei along nerves), but also in multiple types of CNS glia. (D-D″) *IT.0117-Gal4* drives UAS expression in wrapping glia and a small subset of CNS neurons. (E-E″) *WG-SplitGal4* (*nrv2-Gal4^DBD^* and *IT.0117-Gal4^VP16AD^*) drives UAS expression exclusively in wrapping glia.

In *Drosophila,* axons in peripheral nerves are ensheathed by specialized wrapping glia in a manner analogous to vertebrate Remak bundles (Fig. 1A). *Drosophila* larval abdominal nerves contain motor and sensory neuron axons surrounded by multiple glial layers: first the axon-associated wrapping glia, then subperineurial glia, and finally the outermost perineurial glia (von Hilchen et al., 2013; Matzat et al., 2015; Stork et al., 2008) (Fig. 1A). In embryos, axons are initially tightly fasciculated without intervening glial processes. Wrapping progresses such that by the end of the third larval instar (∼3-4 days later) axons are wrapped either individually or in small bundles by wrapping glia membrane (Fig. 1B) (Matzat et al., 2015). This process mirrors vertebrate nerve development when Schwann cells perform radial sorting of axons that are initially tightly fasciculated (Monk et al., 2015). Remak-like multi-axonal ensheathment is thought to represent an ancient form of axon–glial association, and a growing body of evidence demonstrates a high degree of cellular and molecular conservation between ensheathment mechanisms in *Drosophila* and vertebrates (Ghosh et al., 2013; Matzat et al., 2015; Mukherjee et al., 2020; Petley-Ragan et al., 2016; Xie and Auld, 2011).

Axon-associated glia can regulate neuronal development, maintenance and function. Myelination can alter the distribution of axonal proteins, increase axon caliber, and is important for trophic and metabolic support of long axons, although the molecular mechanisms by which myelinating glia perform all of these functions remain incompletely understood (Nave, 2010). Recent work supports the notion that Remak Schwann cells may play similar roles: for example, perturbation of Schwann cell metabolism results in axon degeneration with small caliber, Remak-ensheathed axons degenerating earlier than myelinated ones (Beirowski et al., 2014).

Here, we take advantage of *Drosophila* wrapping glia as a model to identify new molecular regulators of glial ensheathment and support of axons. We demonstrate that wrapping glia are important for normal behavior by developing a Split-Gal4 intersectional driver to ablate larval wrapping glia selectively, which results in severely impaired motor function. Through an RNA interference (RNAi)-based screen, we identify two regulators of ensheathment in larval nerves, the conserved Discoidin domain receptor (Ddr) and its potential ligand, Multiplexin (Mp). Extending our analysis to an adult nerve, we show that loss of glial *Ddr* results in reduced long-term survival of sensory neurons and reduced axon caliber, even without overt effects on ensheathment. These findings establish *Ddr* as an important glial receptor with multiple roles in glia–axon association and signaling.

## RESULTS

### Genetic ablation of wrapping glia impairs larval crawling

To determine whether axonal ensheathment is required for normal axon conduction or circuit function, we sought to ablate wrapping glia selectively. *nrv2-Gal4* drives strong expression of UAS transgenes specifically in wrapping glia without any expression in neurons or other peripheral glia. Although *nrv2-Gal4* is highly specific to wrapping glia in the PNS, it does drive expression in some subtypes of CNS glia, which would complicate the interpretation of behavioral genetic ablation experiments (Fig. 1C-C″). We used a Split-Gal4 intersectional strategy to generate a new wrapping glia-specific driver (Luan et al., 2006). We identified an InSITE collection line, *InSite0117-Gal4* that robustly labeled wrapping glia and a subset of neurons (Fig. 1D-D″) and converted it to *Insite0117-GAL4^VP16AD^* using the InSITE method (Gohl et al., 2011). We recombined this with *nrv2-Gal4^DBD^* (Coutinho-Budd et al., 2017), which resulted in expression of UAS transgenes only where expression of the two hemi-drivers overlaps, i.e. in wrapping glia. No other glia or neurons were labeled by this combination of drivers (Fig. 1E-E″, Fig. S1), which we refer to as *wrapping glia-Split Gal4* (*WG-SplitGal4*).

**Fig. 2.**
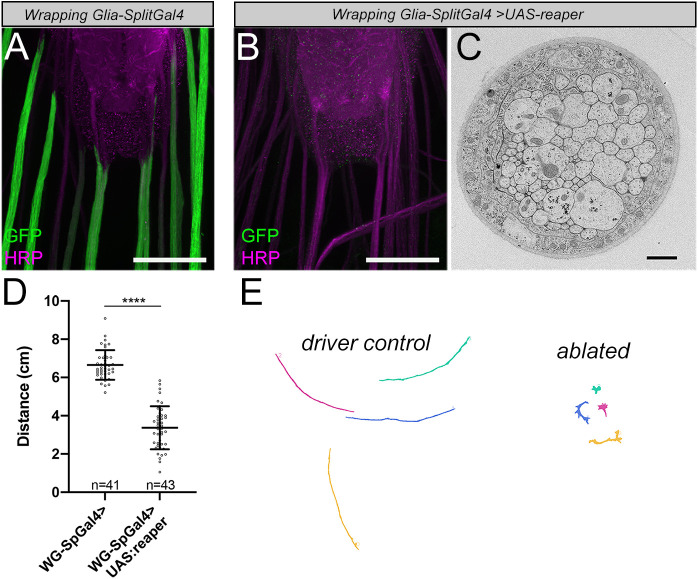
**Genetic ablation of wrapping glia impairs larval crawling behavior.** (A) Expression pattern of *WG-SplitGal4* driving *UAS-CD8:GFP* (green) and HRP counterstain (magenta). Scale bar: 50 µm. (B) Third instar larva with genetically ablated wrapping glia. Note the lack of GFP along nerves. Scale bar: 50 µm. (C) TEM of a nerve from a wrapping glia-ablated animal. Note axons in contact with each other without intervening glial membrane. Scale bar: 1 µm (D) Larval crawling behavior is impaired when wrapping glia are ablated. Unpaired *t*-test, *P*<0.0001. *n*=number of larvae/condition. Error bars represent s.d. (E) Representative crawling paths of control and ablated larvae.

We used *WG-SplitGal4* to drive expression of *UAS-mCD8:GFP* and *UAS-reaper* to ablate wrapping glia. We observed nearly complete loss of larval wrapping glia based on loss of GFP (Fig. 2A,B). We also confirmed loss of wrapping glia using an independent nuclear marker for wrapping glia. The O/E-associated zinc finger protein Oaz (recently identified as the genetic locus of the *Lobe* mutant) was identified as a potential marker for wrapping glia through an enhancer trap screen, prompting us to make an anti-Oaz antibody. Along peripheral nerves that contain nuclei from three subtypes of glia, anti-Oaz only labeled wrapping glia nuclei, which we confirmed using wrapping glia drivers (Fig. S2). After ablation, Oaz nuclear staining was eliminated along nerves that no longer had GFP expression, confirming loss of the cells rather than downregulation of GFP expression (Fig. S3). Furthermore, we used transmission electron microscopy (TEM) to examine the ultrastructure of nerves from animals in which wrapping glia were ablated. In the vast majority of nerves, we saw tightly fasciculated axons without any intervening glial membranes, confirming that wrapping glia had been completely eliminated (Fig. 2C). We did not observe any obvious ingrowth of the outer glia layers between axons that could potentially compensate for the loss of wrapping glia. However, several nerves showed an abnormal hypertrophy of the outer perineurial glia layer (11/49 nerves from five animals; Fig. S3). When we counted the number of axon profiles in each nerve, we found fewer than the expected ∼78 A3-A7 nerves (*w^1118^* average=77.4 axons; wrapping glia-ablated average=63 axons; unpaired *t*-test *P*=0.0006; Fig. S3). This apparent loss of neurons might be due to lack of glial support; however, we cannot rule out the formal possibility that this loss is a secondary effect of glial ablation on neuron differentiation, axon targeting or a response to cell death within the nerve.

We next assayed larval crawling behavior in third instar larvae using the FIMTrack system to record and quantify larval behavior automatically as they crawl along a non-nutritive agar surface (Risse et al., 2017). We found that larvae with ablated wrapping glia crawled significantly less distance than control animals over the 1-min observation period [Fig. 2D,E; mean distance travelled: driver control=6.65±0.77 cm (*n*=41 larvae), ablated=3.37±1.126 cm (*n*=43); *P*<0.0001 unpaired *t*-test]. Traces of crawling paths show that larvae lacking wrapping glia were able to move but failed to have persistent forward motion (Fig. 2E). We also note that larvae lacking wrapping glia had difficulty righting themselves when placed on their dorsal sides and exhibited abnormal postures and body bends, indicating a possible disruption of bilateral or intersegmental coordination caused by wrapping glia ablation. These data provide strong evidence that wrapping glia are required in nerves for normal circuit function.

### Morphology-based RNAi screen identifies *Ddr* as a regulator of wrapping glia development

To identify genes required in glia for normal ensheathment of axons, we conducted a morphology-based RNAi screen: we systematically knocked down genes in wrapping glia and assayed for changes in glial morphology in third instar larval nerves. We used the well-established wrapping glia driver *nrv2-Gal4*, which, within the PNS, is exclusively expressed in wrapping glia, and appeared stronger and more uniform in expression than our driver *WG-SplitGal4* (Stork et al., 2008; Xie and Auld, 2011). We expressed a membrane-bound marker (*UAS-myr:tdTomato*) to visualize wrapping glia morphology and *UAS-RNAi* constructs. In total, we screened a collection of ∼2000 RNAi lines, which comprise the majority of transmembrane, secreted and signaling proteins in the fly genome and included the fly homologs of ∼200 genes strongly expressed in the developing mouse oligodendrocyte lineage based on available RNA-sequencing data (Zhang et al., 2014).

**Fig. 3.**
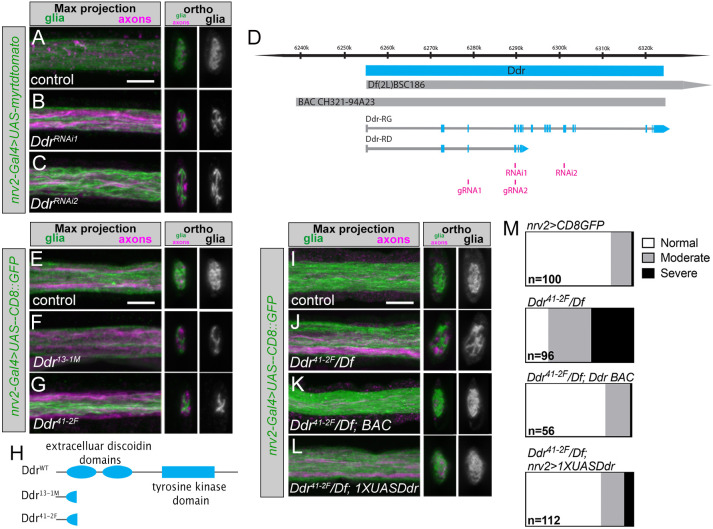
**Ddr is required for normal wrapping glia morphogenesis.** (A-C) Ddr RNAi knockdown with two independent constructs; *nrv2-Gal4*-driven tdTomato is pseudocolored green, Futsch^+^ axons (magenta). (D) The *Ddr* genetic locus: coding region (cyan); *Df(2L)BSC186*; BAC clone for rescue; *Ddr* transcripts RG and RD (exons shown in cyan); RNAi target regions (magenta); and locations of guide RNAs used for the CRISPR-mediated mutagenesis (magenta) are depicted. (E-G) Representative images of *Ddr* homozygous mutants. Compared with control (E), both CRISPR mutant alleles show severe defective glia coverage in nerve cross-sections (F,G). (H) Ddr is a transmembrane receptor tyrosine kinase characterized by extracellular discoidin and discoidin-like domains and an intracellular tyrosine kinase domain. The mutant alleles generated by CRISPR-Cas9 result in truncated peptides without any full domains. (I-L) Representative images of *Ddr* loss-of-function and rescue experiments. (I) *nrv2-Gal4, UAS-CD8:GFP/+* control. (J) *Ddr^41-2F^/DfBSC186* (‘*Ddr* mutant’). This example shows a ‘moderate’ phenotype; more examples can be seen in Fig. S2A. (K) A BAC containing the *Ddr* locus restores normal morphology, as does expression of a *1xUAS-Ddr* construct in wrapping glia (L). (M) Categorical scoring of nerve wrapping glia phenotypes. *n*=number of nerves scored (8-14 larvae per condition). Scale bars: 5 µm.

We examined wrapping glia morphology in the nerve elongation region (NER), defined as the length between where the nerve exits the ventral nerve cord (VNC) to where it touches down in the muscle field. The NER territory is covered by a single wrapping glia cell, termed ePG1, so by looking in the same region ∼200 µm from the distal tip of the VNC, we could examine the same glial cell across nerves and animals. We found that wild-type wrapping glia morphology in third instar larvae includes membrane coverage throughout the interior of the nerve with small gaps through which axons can be seen (Fig. 3A). As a positive control for our screen, we knocked down genes previously shown to be involved in wrapping glia development, including the neuregulin homolog *vein*, the FGF receptor *heartless*, integrin receptors, laminin, and the ceramide synthetase gene *schlank* (Franzdóttir et al., 2009; Ghosh et al., 2013; Kottmeier et al., 2020; Matzat et al., 2015; Petley-Ragan et al., 2016; Xie and Auld, 2011), all of which resulted in defects in axon ensheathment that were easily identifiable at the light level (Fig. S4). Each of these genes have homologs that have been implicated in oligodendrocyte and/or Schwann cell development, further supporting molecular conservation of vertebrate and invertebrate axonal ensheathment mechanisms (Barros et al., 2009; Feltri et al., 2002; Furusho et al., 2009; Lyons et al., 2005; Michailov et al., 2004; Pereira et al., 2009; Stassart et al., 2013; Taveggia et al., 2005; Yu et al., 2009).

Among potential hits for which the function had not previously been studied in the development of either vertebrate or invertebrate glia was the *Discoidin domain receptor* (*Ddr*) gene. *Drosophila Ddr* encodes a cell surface receptor tyrosine kinase homologous to vertebrate Ddr1 and Ddr2. In the screen, knocking down *Ddr* with the *nrv2-Gal4* driver using two independent, non-overlapping RNAi constructs led to altered wrapping glia morphology with the nerve cross-section incompletely covered by glial membrane (Fig. 3A-C). We repeated this experiment using *WG-SplitGal4* and found that this also caused abnormal glia morphology; however, knocking down Ddr using a pan-neuronal driver (*elav-Gal4*) did not alter glia morphology (Fig. S5).

### *Ddr* mutants exhibit defects in axonal ensheathment

To confirm our RNAi findings, we created mutant alleles using a CRISPR-Cas9 strategy. Briefly, using two gRNAs against widely spaced, adjacent exons, we generated fly stocks in which an ∼11 kb region of the *Ddr* coding region was excised near the 5′ end (Fig. 3D). *Ddr* encodes two potential isoforms: a short isoform (380 amino acids) that lacks transmembrane and kinase domains, and a full-length isoform that encodes a 1054 amino acid protein. We identified two independent alleles in which the deletion causes a frameshift to generate early stop codons. These are predicted nulls, as they result in 150 amino acid (*Ddr^13-1M^*) or 134 amino acid (*Ddr^41-2F^*) long peptides lacking all known functional domains (Fig. 3H). Both mutant alleles were homozygous viable and viable when placed in *trans* to large deficiencies that uncover the *Ddr* locus.

We analyzed *Ddr* homozygous mutant animals using fluorescence confocal microscopy and found that wrapping glia morphology was impaired (Fig. 3E-G). We focused our loss-of-function analyses using the combination of *Ddr^41-2F^* and *Df(2L) BSC186*, the shortest deficiency that still deletes the entire *Ddr* coding region (Fig. 3D). We found that animals lacking Ddr had impaired wrapping (Fig. 3I,J,M; control: 79% normal morphology; *Ddr/Df*: 21% normal morphology; see Fig. S6 for examples of categorical scoring). This phenotype is not due to a change in the number of wrapping glia, as the NER we examined is typically covered by a single wrapping glia cell, and this was not changed in *Ddr* mutants (one cell/NER; control: 32/32 nerves, nine animals; *Ddr*: 53/53 nerves, 11 animals). Introduction of a single copy of a BAC clone from the CHORI-322 library (Venken et al., 2009) containing the entire *Ddr* locus and a ∼17.4 kb upstream region into the mutant strain restored normal wrapping morphology (Fig. 3K,M; BAC rescue: 75% normal morphology), as did resupplying Ddr specifically in wrapping glia via a *1xUAS-Ddr* construct driven by *nrv2-Gal4* (Fig. 3L,M; *1xUAS* rescue: 70% normal morphology). This wrapping glia-specific rescue, combined with the results from the *nrv2-Gal4*, *WG-SplitGal4* and *elav-Gal4* RNAi experiments (Fig. 3A-C, Fig. S5), demonstrate that Ddr is required in wrapping glia for their proper morphogenesis. Interestingly, higher level overexpression using a *5xUAS-Ddr* construct caused morphological defects in a control background, which were not observed with the *1xUAS-Ddr* construct, suggesting that Ddr levels may need to be tightly regulated to promote normal wrapping and morphological development (Fig. S7).

**Fig. 4.**
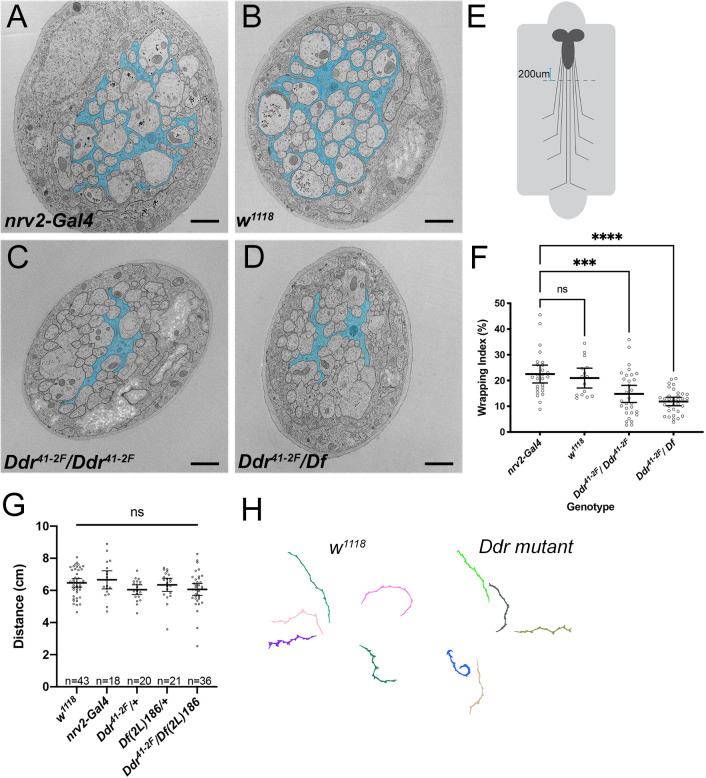
**Loss of *Ddr* impairs axon ensheathment in larval nerves.** TEM cross-sections of an abdominal nerve from third instar larva from each of the following genotypes: (A) control: *nrv2-Gal4, UAS-CD8:GFP/+*; (B) control: *w^1118^*; (C) *Ddr^41-2F^/Ddr^41-2F^; nrv2-Gal4, UAS-CD8:GFP/+*; (D) *Ddr^41-2F^/Df(2L)BSC186; nrv2-Gal4, UAS-CD8:GFP/+*. Wrapping glia are highlighted in cyan. (E) Schematic of larval fillets for TEM. Sections were collected ∼200 µm from the posterior tip of the VNC. (F) Quantification of WI. *nrv2-Gal4, UAS-mCD8:GFP/+* controls (WI: 22.5%, *n*=26 nerves, 5 larvae); *w^1118^* wild-type background strain (WI: 21%, *n*=15 nerves, 3 larvae; *P*=0.9546); *Ddr*^−/−^ (WI: 14.8%, *n*=30 nerves, 4 larvae; ***P=0.0006); *Ddr/Df* (WI: 11.9%,*n*=33 nerves, 4 larvae; ****P<0.0001); one-way ANOVA with Dunnett's multiple comparisons against *nrv2-Gal4*. (G) Larval crawling, as measured by distance traveled per minute in *Ddr* mutants and controls (*nrv2-Gal4, UAS-mCD8:GFP/+* in background of all conditions except *w^1118^*). One-way ANOVA with Tukey's multiple comparisons. *n*=number of larvae/condition. (H) Representative crawling paths. Error bars represent 95% confidence interval. ns, not significant. Scale bars: 1 µm.

We next analyzed nerves from control and *Ddr* mutant animals using TEM and the ‘wrapping index’ (WI) metric to quantify ensheathment defects (Matzat et al., 2015). WI is equal to the number of individually wrapped axons plus the number of small bundles of axons, divided by the total number of axonal profiles, and is expressed as a percentage. In control nerves (Fig. 4A,B), average WI was ∼21% (Fig. 4F; *nrv2* driver control: mean=22.5%, *n*=26 nerves from five larvae; *w^1118^* control: mean=21%, *n*=15 nerves from three larvae), consistent with previous reports (Matzat et al., 2015). We observed less separation of axons by wrapping glia membrane in *Ddr* mutants (Fig. 4C,D): both *Ddr^41-2F^* homozygous and *Ddr^41-2F^*/*Df(2L)BSC186* animals exhibited a significant decrease in wrapping index [Fig. 4F; *Ddr/Ddr* mean=14.8%, *n*=30 nerves from four larvae (*P*=0.0006 compared with *nrv2* control); *Ddr/Df(2L)BSC186*: mean=11.9%, *n*=33 nerves from four larvae (*P*<0.0001 compared with *nrv2* control); one-way ANOVA with Dunnett's multiple comparisons].

To determine whether loss of Ddr disrupted the gross morphology of the nerve, we also assessed the other glial and axonal populations in mutant nerves. We did not observe changes in either subperineurial or perineurial glia in TEM sections in the mutant conditions, or differences in the total number of axon profiles observed (Fig. S8), suggesting that overall nerve assembly was relatively normal. Therefore, loss of Ddr seems to alter wrapping specifically without disrupting nerve morphology.

### Incomplete ensheathment of axons does not alter simple larval crawling behaviors

We next investigated whether impaired ensheathment in *Ddr*^−/−^ larvae was sufficient to impair behavior. We assayed larval crawling behavior in *Ddr* mutant third instar larvae again using the FIMTrack system. Basic crawling behavior (i.e. total distance traveled/minute) was indistinguishable between control and *Ddr* mutant larvae (Fig. 4G,H), in contrast to the strong effects seen when wrapping glia were ablated. These results suggest that the incomplete wrapping observed in *Ddr* mutants is sufficient to support basic neuronal functions during larval stages.

### Ensheathment of axons in an adult nerve is normal in *Ddr* mutants

**Fig. 5.**
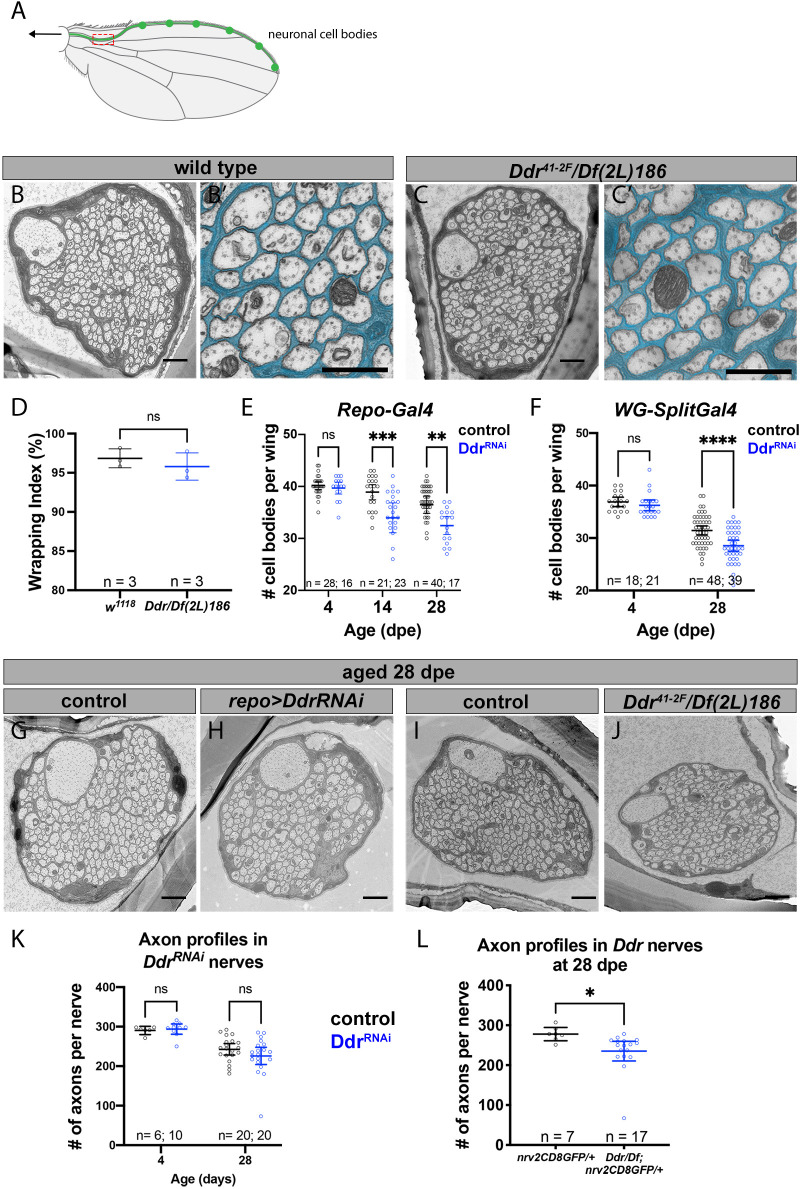
**Loss of *Ddr* impairs long-term neuronal survival without affecting wrapping in adult nerves.** (A) The L1 vein lies along the anterior margin of the wing and contains ∼280 sensory neurons (schematized in green). Axons coalesce in the L1 sensory nerve and project into the CNS (arrow). The TEM sectioning window (red box) contains all sensory neuron axons within the nerve. (B) Cross-section TEM of the L1 nerve at 5 dpe. (B′) Magnified view from B with wrapping glia pseudocolored cyan. (C) Nerve morphology in *Ddr* mutant at 5 dpe. (C′) Magnified view from C with wrapping glia pseudocolored cyan. (D) WI quantification. Unpaired *t*-test, *P*=0.4378. (E) Number of glutamatergic GFP^+^ cell bodies in wings from aged *repo*>*Ddr*^RNAi^ knockdown animals compared with age-matched controls. 4 dpe *P*=0.9774; 14 dpe ***P=0.0007; 28 dpe **P=0.005 (two-way ANOVA with Sidak's multiple comparisons). (F) Number of glutamatergic GFP^+^ cell bodies in wings from aged *WG-SplitGal4* >*Ddr*^RNAi^ knockdown animals compared with age-matched controls. 4 dpe *P*=0.7276; 28 dpe ****P<0.0001 (two-way ANOVA with Sidak's multiple comparisons). (G) Control nerve at 28 dpe. (H) *Ddr^RNAi^* knockdown at 28 dpe. (I) Control nerve from a 28 dpe animal. (J) Representative TEM of a nerve from a 28 dpe *Ddr* mutant. (K) Quantification of axon profile number in control and *Ddr^RNAi^* glial knockdown nerves. 4 dpe *P*=0.9755; 28 dpe *P*=0.2519 (two-way ANOVA with Sidak's multiple comparisons). (L) Quantification of axon profiles from 4 dpe and 28 dpe control and *Ddr* mutants shows a significant reduction in axon number. *P*=*0.0343 (unpaired *t*-test). Error bars represent 95% confidence interval. ns, not significant. Scale bars: 1 µm (B,C,G-J); 600 nm (B′,C′).

Neuronal defects that are secondary to glial dysfunction can be age or stress related in vertebrate animals (Beirowski et al., 2014; Lappe-Siefke et al., 2003; Saab et al., 2016; Yin et al., 1998; Zöller et al., 2008), prompting us to extend our studies to adult flies that can be studied over longer periods. We turned our attention to the L1 sensory nerve in the adult wing. Sensory bristles and campaniform sensilla along the wing edge are innervated by ∼280-290 peripheral sensory neurons located within the L1 wing vein. The axons of these neurons form the L1 sensory nerve that projects into the thorax (Fig. 5A). The organization of the nerve is reported to be similar to larval nerves, with axons ensheathed by wrapping glia (Neukomm et al., 2014). One difference is that there are many more wrapping glia cells along the wing nerve: ∼40 (Fig. S9) compared with just three along the larval abdominal nerves. Axon ensheathment in this nerve had not previously been examined by TEM. We developed a protocol to perform TEM on the adult wing in order to examine the fine details of L1 wing nerve morphology. By imaging in the region distal to the fusing of the L3 nerve but proximal to the first sensory cell body along the anterior wing margin, we could reliably visualize all of the axons present in the L1 nerve and surrounding glia (Fig. 5A, dashed red box). In control nerves at 5 days post-eclosion (dpe), we were surprised to find that all axons appeared to be individually ensheathed by glial membranes and separated from one another (Fig. 5B,B′), instead of persisting in bundles as in larval nerves (compare with Fig. 1A). When we examined *Ddr* mutant wings of the same age, we found no obvious defects in wrapping or axon separation between control and *Ddr* mutant wing nerves (Fig. 5C-D), in contrast to the impaired wrapping we observed in *Ddr* mutant larval nerves. The number of wrapping glia nuclei was unchanged between mutant and control animals along the entire nerve or within our region of analysis (Fig. S9), suggesting that the lack of ensheathment phenotype is not attributable to compensatory wrapping glia proliferation. Ddr may be dispensable for wrapping in the adult, work redundantly with other molecules, be masked by the higher density of wrapping glia naturally in this region, or cause only a transient delay in wrapping that is rectified within a few days of eclosion.

### Loss of Ddr impairs the long-term survival of adult sensory neurons

The lack of overt ensheathment defects in this nerve allowed us to assess whether Ddr might have additional roles in wrapping glia. We proceeded with experiments to determine whether long-term neuronal health in the adult L1 wing nerve might be impacted by loss of Ddr. To test this, we labeled the ∼40 glutamatergic neurons in the wing nerve using the *QF2/QUAS* binary system (*VGlut-QF2; QUAS-6xGFP*) (Potter et al., 2010), while at the same time using *repo-Gal4* to knock down *Ddr* in glia using RNAi. We chose *repo-Gal4* for our initial experiments because it is a strong, consistent driver in adult glia, including wrapping glia. We examined wings at 4, 14 or 28 dpe and counted healthy GFP^+^ neuronal cell bodies along the wing margin at these time points from control and *Ddr-RNAi* animals to determine if loss of Ddr had any impact on long-term neuronal survival. (See Fig. S10 for example images of healthy and unhealthy cell bodies.) The number of healthy neuronal cell bodies at 4 dpe was the same between control and *Ddr* knockdown animals, suggesting that knockdown did not affect neurogenesis (Fig. 5E; 4 dpe mean cell body count=40.2 versus 39.7, *P*=0.9774). Despite beginning with the same number of neurons, we found that there were significantly fewer healthy cell bodies in aged *Ddr*-*RNAi* wings compared with control at both ages examined (Fig. 5E; 14 dpe mean cell body count 38.9 versus 34.0, *P*=0.0007; 28 dpe mean cell body count 36.5 versus 32.5, *P*=0.005; two-way ANOVA with Sidak's multiple comparisons). We repeated these experiments using the *WG-SplitGal4* driver to determine whether these effects were wrapping glia specific. Similar to our results using *repo-Gal4*, we found that, despite beginning with the same number of neurons (Fig. 5F; 4 dpe mean cell body count=36.89 versus 36.24, *P*=0.7276), we found significantly fewer healthy cell bodies in the Ddr knockdown condition at 28 dpe (Fig. 5F; 31.44 versus 28.54, *P*<0.0001; two-way ANOVA with Sidak's multiple comparisons). These results indicate that, despite not overtly disrupting glial ensheathment, knockdown of *Ddr* in wrapping glial cells negatively impacts long-term neuron health and survival.

To extend these findings, we used TEM to visualize cross-sections of L1 nerves from aged control and glial *Ddr-RNAi* animals. To mirror our fluorescence experiments, we used the same genotypes and we first examined wings at 4 dpe. The number of axon profiles in control nerves at 4 dpe showed little variation and was indistinguishable from *repo>Ddr-RNAi* (Fig. 5K; mean axon profile count 290.3±10 versus 293.9±18.3, *P*=0.98). In aged (28 dpe) control and *repo>Ddr* knockdown wing nerves, although wrapping again appeared normal there was overall more variability in axon profile counts (Fig. 5G,H,K: control=242.7±31.02, *Ddr-RNAi*=226±46.5 axon profiles), but there was not a significant difference in axon profile count in the *Ddr-RNAi* condition (Fig. 5K; *P*=0.25; two-way ANOVA with Sidak's multiple comparisons). This is in contrast to our findings in the same genotype when we focused exclusively on *VGlut-QF2*^+^ cell bodies (which marked ∼40 cell bodies out of ∼290 cell bodies total in the L1 nerve). We repeated these experiments using *Ddr* mutant animals and found a small but significant difference in the number of axon profiles (∼15% fewer axons) consistent with the size of our original findings with *VGlut-QF2-*labeled neurons (Fig. 5I,J,L; control=277.9±17.9, *Ddr*=235.2±48.2, *P*=0.022; unpaired *t*-test). Given that the effect was stronger in whole animal mutants, this may reflect insufficient RNAi knockdown, but we cannot rule out that loss of Ddr from neurons, or another tissue, might contribute to this effect, or that only specific subsets of neurons are particularly vulnerable to Ddr loss. Nevertheless, all together these data indicate that glial Ddr modulates the long-term health of sensory neurons.

### Glial Ddr promotes increased axon caliber

**Fig. 6.**
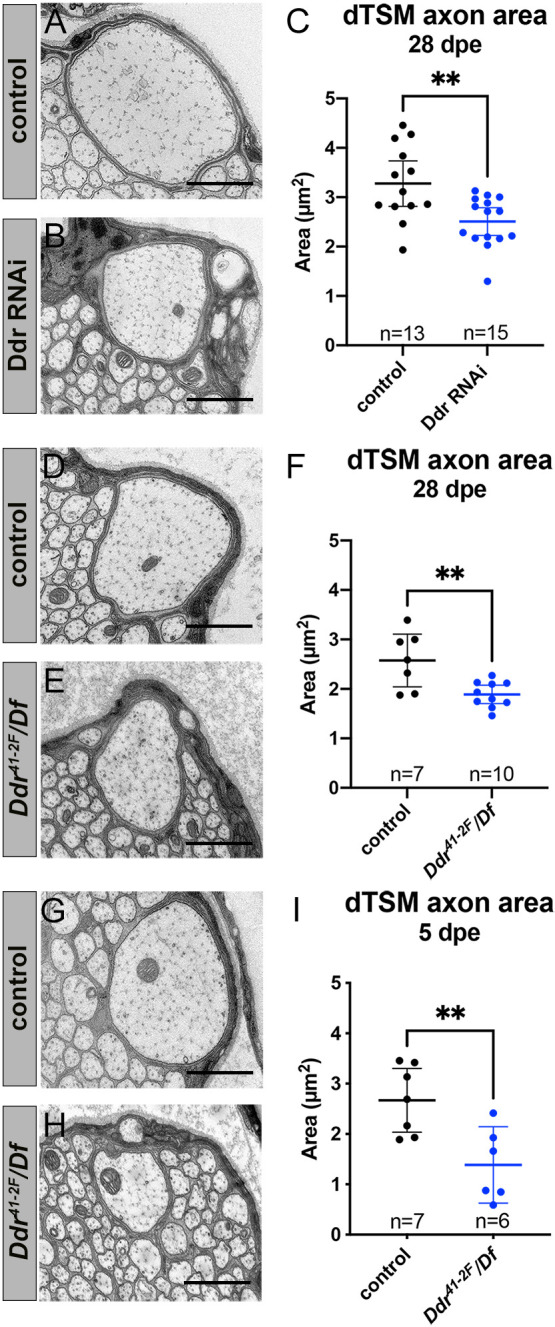
**Ddr is required for normal axon caliber growth.** (A-C) Axon caliber of dTSM axons as measured by the cross-sectional area in TEM images in 28 dpe *Ddr^RNAi^* nerves compared with age-matched controls. **P=0.0037 (unpaired *t*-test). (D-F) Axon caliber of dTSM axon in 28 dpe *Ddr* loss-of-function nerves compared with age-matched controls. **P=0.0044 (unpaired *t*-test). (G-I) Axon caliber of dTSM axons in 5 dpe *Ddr* loss-of-function nerves compared with age-matched controls. **P=0.0073 (unpaired *t*-test). Error bars: 95% confidence interval. Scale bars: 1 µm.

Myelination can induce many changes in underlying axons, including the redistribution of axonal proteins and increased axon caliber (Stassart et al., 2018), but whether non-myelinating ensheathment has similar effects is less clear. TEM of the L1 nerve revealed the presence of a single, prominent, large-caliber axon (Fig. 5B). This axon is immediately identifiable in every sample, allowing us to compare its features across animals and conditions. A survey of the literature revealed that this axon belongs to the distal twin sensilla of the margin (dTSM) neuron (Dickinson and Palka, 1987). A striking but unexpected observation was that the dTSM axon in *Ddr* mutants was smaller than in controls, although it was still clearly identifiable as the largest axon in the nerve. We quantified axon caliber by measuring the cross-sectional surface area of this uniquely identifiable axon and comparing appropriate controls to *Ddr* mutant or glial *Ddr-RNAi* conditions from our 28-day-old wing TEM images. We found that in both glial-specific *Ddr* knockdown and *Ddr* whole animal mutant nerves the caliber of the dTSM axon was significantly reduced compared with controls, revealing a role for glial Ddr in modulating axonal caliber even when ensheathment appears normal (Fig. 6A-F).

To determine whether reduced caliber was due to reduced growth versus shrinkage, we examined dTSM caliber in control and *Ddr* mutant wings from 5 dpe and found that the reduction in size is even greater in younger animals (Fig. 6G-I). dTSM axon caliber is reduced by ∼48% in *Ddr* mutants at 5 dpe (mean cross-sectional area: control=2.670 µm^2^ and *Ddr*=1.387 µm^2^) and ∼27% at 28 dpe (mean cross-sectional area: control=2.575 µm^2^ and *Ddr*=1.890 µm^2^). We have examined a small number of L1 nerves from wild-type freshly eclosed flies (<24 h post eclosion) and observed that all axons appear smaller than those from our standard 5 dpe time point, suggesting that the first few days post-eclosion are an important period of axon growth and maturation (Fig. S11). Together, these data suggest that the reduced caliber we see in *Ddr* mutants is due to impaired growth, rather than shrinkage. Because knockdown of *Ddr* selectively in glia is sufficient to cause a similar reduction in caliber to that observed in whole animal mutants (∼23% reduction at 28 dpe in RNAi experiments), our data identifies a non-cell-autonomous role for glial Ddr in the control of axon caliber.

In light of the above findings, we decided to analyze axon caliber in our larval TEM experiments more broadly. We cannot directly compare the axons of uniquely identifiable neurons across animals, as with dTSM, but we can look at the overall distribution of axon sizes between conditions. We found that distributions of axon calibers were significantly different with median axon caliber reduced in *Ddr* mutant nerves relative to controls (median cross-sectional area: control=0.085 µm^2^ and *Ddr*=0.062 µm^2^; *P*<0.0001; Kolmogorov–Smirnov test). We found similar results in wrapping glia-ablated nerves compared with controls (median cross-sectional area: control=0.083 µm^2^ and ablated=0.064 µm^2^; *P*<0.0001; Kolmogorov–Smirnov test). This appears to be mainly attributable to an increased frequency of very small caliber axons in mutant or ablated conditions (Fig. S12). Together, these data support a role for *Ddr* in positively regulating axon caliber at all stages, with the adult data revealing that this function is at least partially independent of its role in ensheathment.

### *Ddr* interacts with *Mp* to promote wrapping in larval nerves

**Fig. 7.**
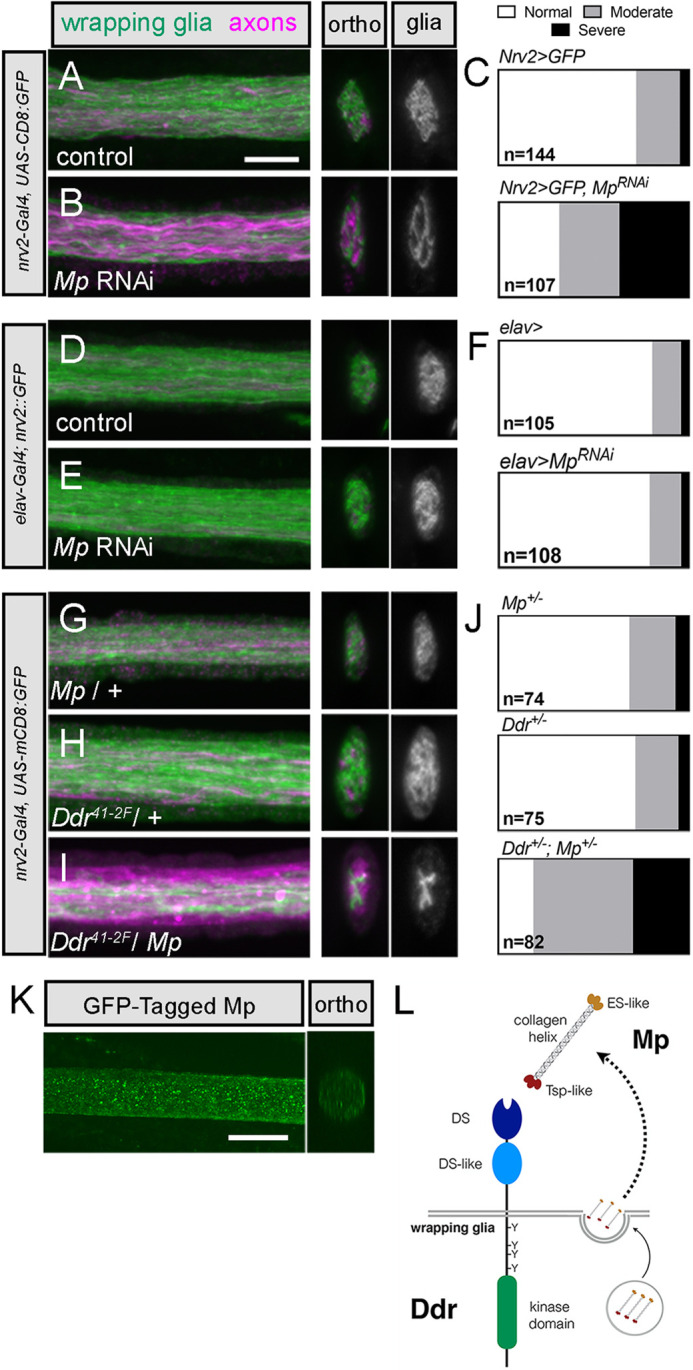
***Mp* genetically interacts with *Ddr* to promote wrapping in larval nerves.** (A-C) Knockdown of Mp in wrapping glia using *nrv2-Gal4* disrupts wrapping glia morphology. *nrv2-Gal4*-driven mCD8:GFP, green; Futsch^+^ axons, magenta. (D-F) Knockdown of Mp in neurons using *elav-Gal4* does not affect wrapping glia morphology, visualized using the *nrv2::GFP* trap. (G-J) Wrapping glia morphology in *Mp/+* and *Ddr/+* nerves, compared with *Mp/+; Ddr/+*. (C,F,J) Categorical scoring of normal, moderately disrupted, and severely disrupted wrapping glia morphology. (K) A MiMiC construct that adds an in-frame GFP tag to Mp protein is localized throughout larval nerves. (L) Proposed model of Mp and Ddr interaction. A single Ddr protein is depicted in the wrapping glia membrane with extracellular discoidin (DS) domains and an intracellular kinase domain. Mp is depicted with its central collagen domain and cleavable endostatin-like (ES-like) and thrombospondin-like (Tsp-like) domains. Based on the strong phenotype observed when Mp is knocked down specifically in wrapping glia, and the lack of phenotype when knocked down in neurons, we hypothesize that wrapping glia secrete Mp, which can act as autocrine activator of Ddr to drive normal wrapping glia morphogenesis. *n*=number of nerves analyzed from 9-13 larvae per condition. Scale bars: 5 µm.

We sought to understand the molecular mechanism(s) by which Ddr is activated to promote axon ensheathment. Mammalian Ddrs are activated by collagens *in vitro* and are thus considered non-canonical collagen receptors (Vogel et al., 1997). We wondered whether *Drosophila* Ddr also interacts with collagens to mediate its effects on wrapping in the larvae. The *Drosophila* genome contains three collagen genes. One of them, *Multiplexin* (*Mp*), was identified as a potential hit that affected wrapping glia morphology in third instar nerves in our initial screen. Knockdown of *Mp* in wrapping glia using *nrv2-Gal4* caused moderate to severe wrapping defects when analyzed by confocal microscopy (72% normal morphology in control versus 32% normal morphology in Mp^RNAi^; Fig. 7A-C). Conversely, knockdown of *Mp* in neurons using the pan-neuronal driver *elav-Gal4* did not affect wrapping glia morphology (81% normal morphology in control versus 80% normal morphology in Mp^RNAi^; Fig. 7D-F). We assessed expression of Mp protein in wild-type nerves using a MiMiC-GFP line in which the endogenous Mp protein is tagged with GFP. We observed punctate GFP expression throughout larval nerves (Fig. 7K), as previously reported (Wang et al., 2019), suggesting that Mp, a secreted protein, is expressed by one or more of the cell types within the nerve, consistent with it having a role in wrapping glia morphogenesis.

To determine whether *Ddr* and *Mp* might be working in the same genetic pathway to modulate wrapping, we crossed heterozygous *Ddr* and *Mp* mutants together and analyzed the double-heterozygous progeny for defects in larval wrapping glia morphology. Although animals that were heterozygous for either gene alone did not show defects in wrapping glia morphology, we found that the double-heterozygous animals exhibited moderate to severe defects in glial morphology (*Ddr/+*: 77% normal morphology; *Mp/+*: 72% normal morphology; *Ddr/Mp*: 18% normal morphology; Fig. 7G-J). These data suggest a strong genetic interaction between the *Ddr* and *Mp* loci and imply they might function in a common genetic pathway to control wrapping glia morphological development. Given that RNAi knockdown of *Mp* specifically in wrapping glia is sufficient to cause a wrapping defect, our data suggest a model in which Mp acts in an autocrine fashion to activate Ddr and drive downstream signaling to promote wrapping (Fig. 7L).

## DISCUSSION

Non-myelinating ensheathment of axons is a conserved but understudied feature of the PNS. Although this type of multi-axonal ensheathment has been less studied compared with myelination, a growing body of evidence indicates it is important for the health and function of neurons and axons in the periphery. For example, Schwann cell-specific loss of the transmembrane receptor LDL receptor related protein-1 (LRP1) causes both thin myelin and abnormal Remak bundle structure. These conditional knockout animals also showed a lowered pain threshold, suggesting that the physiology of nociceptor neurons is impaired when Remak ensheathment is disrupted (Orita et al., 2013). Disrupting metabolism in Schwann cells causes progressive axon loss, with small unmyelinated fibers dying first, before myelinated fibers begin to show signs of degeneration (Beirowski et al., 2014; Viader et al., 2011, 2013). In the fly, disruption of axonal wrapping leads to uncoordinated behavioral responses that hint at aberrant ephaptic coupling between neighboring axons in nerves when not properly separated (Kottmeier et al., 2020). Such coupling could cause the inappropriate activation of sensory or nociceptive neurons underlying peripheral neuropathies. Previous studies from our lab have shown that wrapping glia are required to clear neuronal debris after nerve injury and mediate injury signaling between injured and intact ‘bystander’ neurons, which might be important for functional recovery after nerve trauma (Hsu et al., 2021; Neukomm et al., 2014). These and other findings suggest that Remak-type ensheathment and axon–glia signaling of unmyelinated fibers play a variety of underappreciated roles in peripheral nerve physiology that contribute to the pathophysiology of a number of PNS disorders, including debilitating peripheral neuropathies and responses to nerve injury.

To gain insight into non-myelinating ensheathment, we used the *Drosophila* peripheral nerves to identify a molecular pathway important for the development and function multi-axonal ensheathment. We generated a new Split-Gal4 intersectional driver to target wrapping glia more specifically for functional and behavioral studies in order to improve our understanding of whether and how wrapping glia support axon health, physiology and, ultimately, circuit function. Finally, we uncovered roles for glia in mediating long-term neuronal survival and driving increased axon caliber that are separable from overt effects on wrapping, demonstrating that non-myelinating ensheathing glia perform crucial, previously unappreciated, roles in nervous system development, maintenance and function.

### Ddr and Mp regulate wrapping in larval nerves

A main advantage of *Drosophila* is the ability to conduct large-scale *in vivo* screens. We made use of available *UAS-RNAi* libraries to carry out a broad screen for regulators of axonal ensheathment in intact nerves. Our morphological screen was sensitive enough to identify genes previously implicated in wrapping glia development, including *vn*, *LanB1* and *mys*, validating our approach. Moreover, in the case of *Ddr*, we were able to identify an important regulator of ensheathment that a simple behavioral or lethality screen would have missed in light of our follow-up behavioral testing. Knockdown of *Ddr* in wrapping glia resulted in reduced glial membrane coverage in nerve cross-sections by fluorescence microscopy. Similar phenotypes were observed in *Ddr* loss-of-function animals and could be rescued by resupplying Ddr specifically in wrapping glia, confirming the specificity of our RNAi results. TEM clearly showed that reduced glial membrane coverage at the light level corresponds to decreased axon wrapping.

Although neither of the vertebrate homologs, Ddr1 and Ddr2, has been explicitly implicated in glial development, several lines of evidence suggests that Ddr1 may have a conserved role in vertebrate glial development or function. Ddr1 is highly expressed in the mouse oligodendrocyte lineage starting from when the cells begin to associate with axons, is upregulated in newly formed oligodendrocytes after cuprizone treatment, and is expressed in both myelinating and Remak Schwann cells (Franco-Pons et al., 2006, 2009; Gerber et al., 2021; Zhang et al., 2014). Moreover, DDR1 is expressed in human oligodendrocytes and myelin, and variants in the human gene have been correlated with abnormal white matter and schizophrenia (Gas et al., 2018; Roig et al., 2010).

Vertebrate Ddr1 and Ddr2 are potently activated by collagens *in vitro* (Vogel et al., 1997), prompting us to investigate whether collagens were involved with Ddr function in fly nerves. We found that knockdown of the *Drosophila* collagen Mp specifically in wrapping glia but not in neurons disrupted ensheathment. Together with the established roles for vertebrate Ddr1 and Ddr2 as collagen receptors, the strong genetic interaction we observed between *Ddr* and *Mp* is consistent with a model in which Mp acts as a collagen ligand for Ddr during axonal ensheathment. Although the *Mp-GFP* protein trap shows diffuse Mp expression throughout the nerve, it remains unclear precisely which cell type(s) within the nerve are producing it. Previous reports indicate that Mp can be expressed in the outer peripheral glia layers (Wang et al., 2019), so they may provide some Mp to the wrapping glia. However, the strong ensheathment defect seen when *Mp* is knocked down exclusively in wrapping glia indicates that wrapping glia themselves are likely to be the primary, relevant source of the Mp required for their own morphogenesis. Schwann cells similarly rely on components of their own basal lamina to regulate their development. For example, laminin-211 serves as a ligand for GPR126 to promote myelination (Petersen et al., 2015). Mp is the sole *Drosophila* homolog of collagen types XV/XVIII, containing a central helical collagen region with a cleavable N-terminal thrombospondin-like domain and C-terminal endostatin-like domain (Meyer and Moussian, 2009; Myllyharju and Kivirikko, 2004). Collagen 15a1 and 18a1 are expressed in mouse peripheral nerves and *Col15a1* mutants have radial-sorting defects, suggesting that the role of *Mp* in promoting axon wrapping is likely conserved (Chen et al., 2015; Gerber et al., 2021; Rasi et al., 2010). In fact, *Mp* appears to play multiple roles in nerve biology. For example, Mp secreted by the outer glia layers acts via its cleaved endostatin domain to modulate homeostatic plasticity at motor neuron synapses (Wang et al., 2014; Wang et al., 2019). How Ddr activation within wrapping glia ultimately drives axon wrapping still remains to be determined, but Ddr joins two other receptor tyrosine kinases – EGFR and FGFR – as important and conserved regulators of axon ensheathment (Franzdóttir et al., 2009; Kottmeier et al., 2020; Matzat et al., 2015). As a non-canonical collagen receptor, Ddr may also interact with other collagen receptors, such as integrins (known to play roles in wrapping glia development; Xie and Auld, 2011), to sense and remodel the extracellular matrix and permit extension of glia processes between axons, similar to its roles in promoting tumor metastasis (Itoh, 2018).

### Wrapping glia are required for normal larval behavior

The *nrv2-Gal4* driver has been the standard method to genetically target wrapping glia for morphological studies, but it is imperfect for manipulation of wrapping glia in ablation or behavioral assays owing to its expression in several subtypes of CNS glia. We generated a new Split-Gal4 intersectional driver that drives exclusively in wrapping glia. This allowed us to perform precise ablation of wrapping glia that led to severely impaired larval locomotion, indicating that the wrapping glia are essential for basic crawling circuit function. This phenotype was particularly striking in light of that fact that we did not observe any clear crawling defect in *Ddr* mutant larvae, even though wrapping was severely impaired. It may be possible that non-contact-mediated mechanisms, such as one or more secreted factors, constitute the essential contribution of wrapping glia to axon health and physiology. Alternatively, perhaps even a small amount of direct glia–axon contact may be sufficient to support neuron health and axon function. This would be consistent with the lack of overt behavioral defects in newly hatched first instar larvae, which have poor wrapping compared with later stages (Matzat et al., 2015; Stork et al., 2008), and even in wild-type third instar larvae, in which not every axon is individually wrapped. It is also consistent with our findings that many nerves in WG-ablated larvae seem to be missing axons, whereas we did not observe this in Ddr mutant nerves. Our results are also similar to what has been recently reported (Kottmeier et al., 2020) using a different approach to ablate wrapping glia, where only minor behavioral defects were observed upon FGFR signaling disruption but profound crawling defects were seen upon ablation. As with all ablation studies, we cannot strictly rule out unexpected negative side effects of the ablation itself; however, using a genetic approach should limit collateral damage (compared with laser or toxin approaches). Together, these data support the conclusion that even limited wrapping or simply some degree of glia–axon contact is sufficient to support axon survival and nerve function compared with no glia at all at least for the first ∼5 days of larval life.

### Loss of Ddr impairs long-term neuronal survival

Previous studies of oligodendrocytes and Schwann cells have found that impairing glial function can result in seemingly normal wrapping and circuit function in young animals, with deficits only appearing when the system is stressed or aged (Beirowski et al., 2014; Lappe-Siefke et al., 2003; Saab et al., 2016; Zöller et al., 2008). Studying wrapping in adult *Drosophila* allows for aging and maintenance studies that the short larval period precludes. Adult peripheral nerves are encased in a transparent but hard cuticle that allows for live imaging but makes fixation challenging. Because of the resolution limits of light microscopy, we established a reliable method to study their ultrastructure using TEM. We found that ensheathment in the adult wing nerve differs from that of the larva, as all axons appear to be separated by glial membranes. To our surprise, wrapping was not obviously impaired in adult nerves of *Ddr* knockdown or mutant animals. One difference between larval and adult wrapping glia is the territory size of each cell. In larvae, one wrapping glia cell covers the majority of the nerve from the VNC to the muscle field. This wrapping glial cell must therefore undergo tremendous growth to keep up with nerve elongation as the animal grows, as well as radial growth to ensheathe axons. A single cell can end up covering from ∼750 µm to 2.5 mm of nerve length, depending on the segment, whereas in the wing there are ∼13 wrapping glia along the region of the L1 nerve we analyzed, which is ∼400 µm long. In larval wrapping glia, there are three receptor tyrosine kinases (EGFR, the FGFR Heartless, and now Ddr) that are each required for normal ensheathment, and thus cannot fully compensate for one another (Kottmeier et al., 2020; Matzat et al., 2015). We hypothesize that in the larva the cell is pushed to its growth limits and any perturbation in pro-wrapping signaling has a strong effect on morphology, whereas in the adult nerve the system is robust and redundant enough to withstand perturbations of single genes. Future studies of double and triple mutants may be able to test this hypothesis.

Loss of *Ddr* led to an increase in spontaneous neurodegeneration in the nerve as animals naturally aged. Such an uncoupling of neuron health from overt effects on myelination has been demonstrated previously. For example, *Cnp1* (*Cnp*) mutant mice show severe age-dependent neurodegeneration, although they have grossly normal myelin with only subtle changes in myelin ultrastructure (Lappe-Siefke et al., 2003; Snaidero et al., 2017). Loss of the proteolipid PLP results in axon degeneration despite having largely normal myelin (Garbern et al., 2002; Griffiths et al., 1998; Klugmann et al., 1997). We found that the number of VGlut^+^ neurons was reduced in aged wings of *Ddr* knockdown animals, indicating that wrapping glial *Ddr* is important for long-term neuronal survival. When we analyzed *Ddr* whole animal mutants by TEM we found a small but significant reduction in axon profile number, which should correspond to the number of surviving neurons. Together with the increased variability observed, this suggests that absence of Ddr signaling increases the susceptibility of subpopulations of neurons to insult or injury that may underlie age-related degeneration.

### Glial Ddr regulates axon caliber

Myelination can directly affect the structure and function of the axons they wrap, including controlling caliber. In general, myelination increases caliber. For example, dysmyelinated *Trembler* mice have reduced axon calibers compared with controls (de Waegh et al., 1992), and in the PNS caliber along a single axon can vary with reduced caliber at points without direct myelin contact, such as nodes of Ranvier (Hsieh et al., 1994). Axon caliber is an important determinant of conduction velocity but varies widely between neuronal subtypes, so achieving and maintaining appropriate caliber is crucial for proper circuit function. How non-myelinating ensheathment impacts axon caliber is not understood. Here, we find glial Ddr promotes increased axon caliber. We focused on dTSM, so we could directly compare the caliber of an identifiable axon between conditions. The reductions in caliber were similar between *Ddr* mutants and glial-specific *Ddr^RNAi^*, supporting a non-cell-autonomous role for glial Ddr in regulating axon caliber. The effect is considerable: nearly a 50% reduction in axon caliber at 5 dpe. We hypothesize that by this time point, wild-type dTSM axons have reached their mature caliber, as it is comparable between 5 dpe and 28 dpe in comparable genetic backgrounds. In *Ddr* mutants, however, we observe that the relative size compared with controls changes over time, suggesting that in *Ddr* mutants (or knockdowns) the axon continues to increase its caliber, perhaps in an effort to achieve the optimal size, although the axons still remain ∼25% smaller than wild-type axons at 28 dpe.

Two proteins, MAG, which acts to increase the caliber of myelinated axons (Yin et al., 1998), and CMTM6, which restricts the caliber of myelinated and unmyelinated axons (Eichel et al., 2020), are the only proteins reported to non-cell-autonomously affect the caliber of vertebrate axons, and both do so without overtly affecting myelin. In the fly, we and others have shown a shift in the average size of axons in larval nerves when wrapping glia are absent or severely disrupted, supporting a general role for wrapping glia in promoting axon size (Kottmeier et al., 2020). In the adult, we show that Ddr is still required for increased axon caliber even when wrapping appears intact. The exact molecular mechanism by which Ddr may promote increased caliber size remains unclear as the control of axon caliber, generally, is not well understood. Genes involved in the general regulation of cell size have been implicated as cell-autonomous determinants. For example, in the fly, S6 kinase signaling is a positive regulator of motor neuron size, including axon caliber (Cheng et al., 2011). In mammalian axons, the phosphorylation state of neurofilaments and microtubules determines their spacing to determine caliber (Yin et al., 1998). Determining how glial Ddr activity ultimately influences the axonal cytoskeleton is an important next step. A 25-50% reduction in caliber would be predicted to impact conduction velocity along the dTSM axon. Given that campaniform sensilla provide essential rapid sensory feedback to fine-tune movement, it will be of interest to test conduction velocity and flight behavior in *Ddr* mutant animals to see how the proprioceptive circuit might be affected.

Taken together, our studies identify Ddr as an important regulator of wrapping glia development and function in the fly, with distinct roles in larval and adult wrapping glia. Ddr is essential for the normal morphological development of axon wrapping in the larvae, and also mediates important axon–glia communication that controls axon caliber growth and affects neuronal health and survival. Given its expression pattern in vertebrate oligodendrocytes and Schwann cells, it seems likely that these essential functions are conserved in vertebrates. Further study into how Ddr functions in both fly and vertebrate glia promises to increase our understanding of axon ensheathment in health and disease.

## MATERIALS AND METHODS

### *Drosophila* genetics

*Drosophila melanogaster* were raised using standard laboratory conditions. RNAi experiments were conducted at 29°C, all others at 25°C. A complete list of all fly strains used in this study can be found in Table S1. The full genotypes of animals used in each figure can be found in Table S2. Fly strains generated in this study include *Ddr^41-2F^* and *Ddr^13-1M^*, which were generated using CRISPR-Cas9 editing; *WG-SplitGal4*, which was generated using the InSITE Method (Gohl et al., 2011), and *5XUAS-Ddr* and *1XUAS-Ddr*, which were generated using standard cloning methods*.* See Supplementary Materials and Methods for further details on their construction. These stocks are available upon request.

### Immunohistochemistry and confocal analysis

For confocal analysis, larvae were filleted on Sylgard-coated plates at room temperature in PBS and fixed on a shaker in 4% paraformaldehyde (Electron Microscopy Sciences) in PBS for 20 min, washed with PBS and PBST (0.3% Triton X-100) and incubated in primary antibodies overnight at 4°C. Secondary antibodies were incubated overnight at 4°C in the dark. Samples were mounted on glass slides using Vectashield (Vector Laboratories). Primary antibodies were: chicken anti-GFP (1:1000, Abcam, ab13970); rabbit anti-DsRed (1:600, Clontech, 632496); rat anti-mCherry (1:2000, Invitrogen, 16D7); mouse anti-Futsch (22C10, 1:500, DSHB); mouse anti-Repo (1:100, DSHB); goat anti-HRP pre-conjugated to Alexa Fluor 488, Cy3 or Cy5 (1:100, Jackson ImmunoResearch); rabbit anti-Oaz (1:5000, this study; see Supplementary Materials and Methods and Fig. S2 for further details). Donkey secondary antibodies conjugated to DyLight 488, Alexa Fluor 488, Cy3, Rhodamine Red-X, Cy5, or DyLight 405 were used according to the manufacturer's instructions (Jackson ImmunoResearch). Phalloidin-iFluor 647 and 405 were used at 1:2500 (Abcam). Images were collected on either an Innovative Imaging Innovations (3i) spinning-disk confocal microscope with SlideBook software or a Zeiss spinning disk confocal microscope with Zen software using 10× air, 40×1.3 NA oil or 63×1.4 NA oil objectives. Confocal stacks taken to analyze wrapping glia morphology in nerve cross-sections were taken with the 63× objective, using the recommended optimal slice size of 0.27 μm.

To score wrapping glia morphology, images were obtained ∼200 μm posterior to the VNC (to roughly correspond to the TEM analysis location). At 63× magnification, ∼100 of nerve length could be analyzed in an image. Orthogonal sections were visualized using SlideBook or Zen software and the cross-section of each nerve was examined over the length in the captured image. Morphology was scored categorically as being ‘normal’, having ‘moderate’ defects in nerve coverage, or having ‘severe’ defects in nerve coverage, as indicated in Fig. S6. *n* values are given in figure legends.

### Electron microscopy

Third instar larvae were manually processed for TEM as dissected fillets, using standard TEM processing procedures. *Drosophila* wings were processed using microwave-assisted fixation with a protocol adapted from previous studies (Cunningham and Monk, 2018; Czopka and Lyons, 2011), with elimination of all 10 min room temperature ‘hold’ steps. Samples were embedded in EMbed 812, 70 nm sections were collected on 200-mesh copper grids (larvae) or 100-mesh Formvar film-coated grids (wings), counterstained with uranyl acetate and lead citrate and imaged on a Tecnai T12 electron microscope equipped with an AMT digital camera and software. See Supplementary Materials and Methods for step-by-step details on the fixation protocol.

### Aging assay and live imaging of wings

Animals were aged at 25°C in cornmeal agar vials for the indicated number of days, with transfers to fresh food vials every 3-7 days. Flies were anesthetized, wings removed, mounted and imaged as described by Neukomm et al. (2014) 4, 14 or 28 days after eclosion. For the neuronal survival assays, wings were inspected for tears or injuries along the L1 vein with transmitted light and only wings with no visible physical damage were assessed further. GFP^+^ neurons were counted as intact if they had a clear nucleus and dendrite or were considered dead if they were shrunken and the dendrite or nucleus were not clearly visible, as shown in the example images in Fig. S10.

### Larval tracking

Larval crawling behavior was analyzed using the frustrated total internal reflection imaging method and FIMTrack software (Risse et al., 2017). Briefly, larvae were washed briefly in ddH20 and kept on agar plates prior to testing. Approximately five larvae of the same genotype were placed near the center of a 0.8% agar surface positioned above an IR camera using a paintbrush. Behavior was recorded at room temperature at 10 frames/s for 1 min. Videos were analyzed using FIMTrack software (Risse et al., 2017) to extract data about individual larva. Crawling paths for each larva were automatically generated by FIMTrack and information of total distance travelled for each larva was exported to Excel and converted from pixels to cm before being analyzed using GraphPad Prism software. Only larvae that were successfully tracked for the full minute were included in the analysis.

### Analysis and statistics

TEM image analysis was performed in Fiji/ImageJ using an AMT plugin. WI was quantified as described by Matzat et al. (2015). Briefly, the Cell Counter plugin was used to manually tally the total number of axons and then manually tally the number of bundles or singly wrapped axons. WI was then computed for each nerve as: number of singly wrapped axons+number of axon bundles/total number of axons, expressed as a percentage. Axon counts in wing nerves were also performed manually with the Cell Counter plugin in Fiji. Axon cross-sectional area calculations were performed using the polygon selection tool to trace the circumferences of axons manually, followed by measurement of area. Nerves were excluded from an analysis if knife marks, resin folds, or debris blocked the feature(s) needing measurement, or if axonal profiles were not clear (as a result of being cut at an angle or poor fixation). Size comparisons were only made from images taken at the same magnification. For the analysis of axon size in wrapping glia-ablated larval nerves, we limited our analysis to nerves that had at least 70 axon profiles. For most experiments, both male and female animals were used unless the genetics of a specific experiment only allowed for one sex to be used. Only female flies (wings) were used for adult TEM analysis. Statistical analysis was performed using GraphPad Prism software. The appropriate statistical test was determined based on the experimental design, results of the D'Agostino–Pearson normality test, and how many conditions were being compared. Details on the exact sample sizes and statistical tests used for each experiment are included in the corresponding Results section and/or figure legend.

## Supplementary Material

10.1242/develop.200636_sup1 Supplementary information
