# Supplementary material for: Discoidin domain receptor regulates ensheathment, survival and caliber of peripheral axons
Source: Development. 2022 Dec 13;149(23):dev200636. doi: 10.1242/dev.200636 (PMC10112903; doi:10.1242/dev.200636)
Supplement: Supplementary information [file develop-149-200636-s1.pdf]

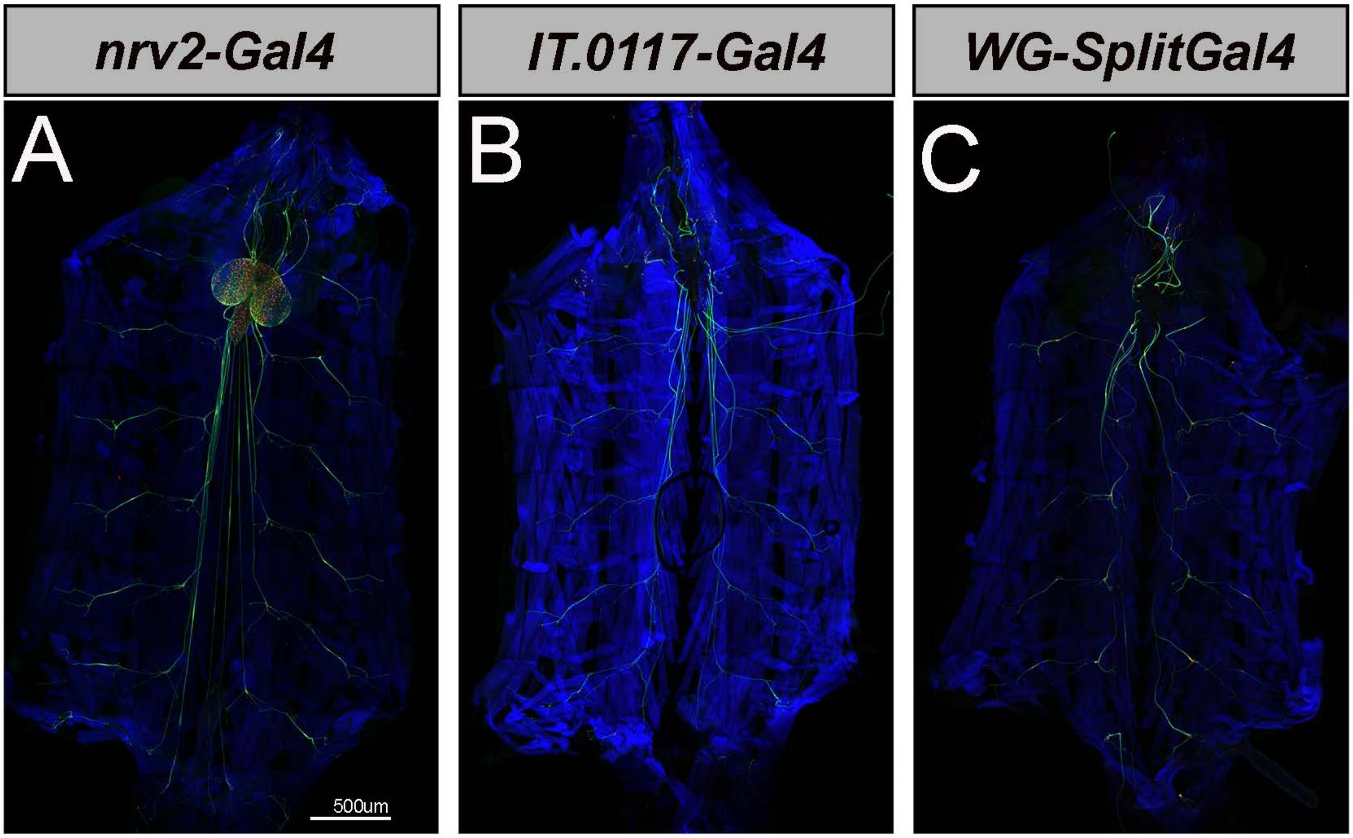

Fig. S1. ***WG-SplitGal4* whole body expression pattern**

(A) *nrv2-Gal4* driving *UAS-CD8:GFP* (green) and *UAS-mCherry*^{nls} (red). Body wall muscles are labelled with phalloidin (blue).

(B) *IT.0117-Gal4* driving *UAS-myrGFP* (green) and *UAS-H2B:mCherry* (red).

(C) *WG-SplitGal4* driving *UAS-mCD8:GFP* (green) and *UAS-mCherry*^{nls} (red). *WG-SplitGal4* drives exclusively in wrapping glia in the periphery without any evidence of neuronal or glial expression in the CNS nor in any other tissue in the larva.

Development • Supplementary information

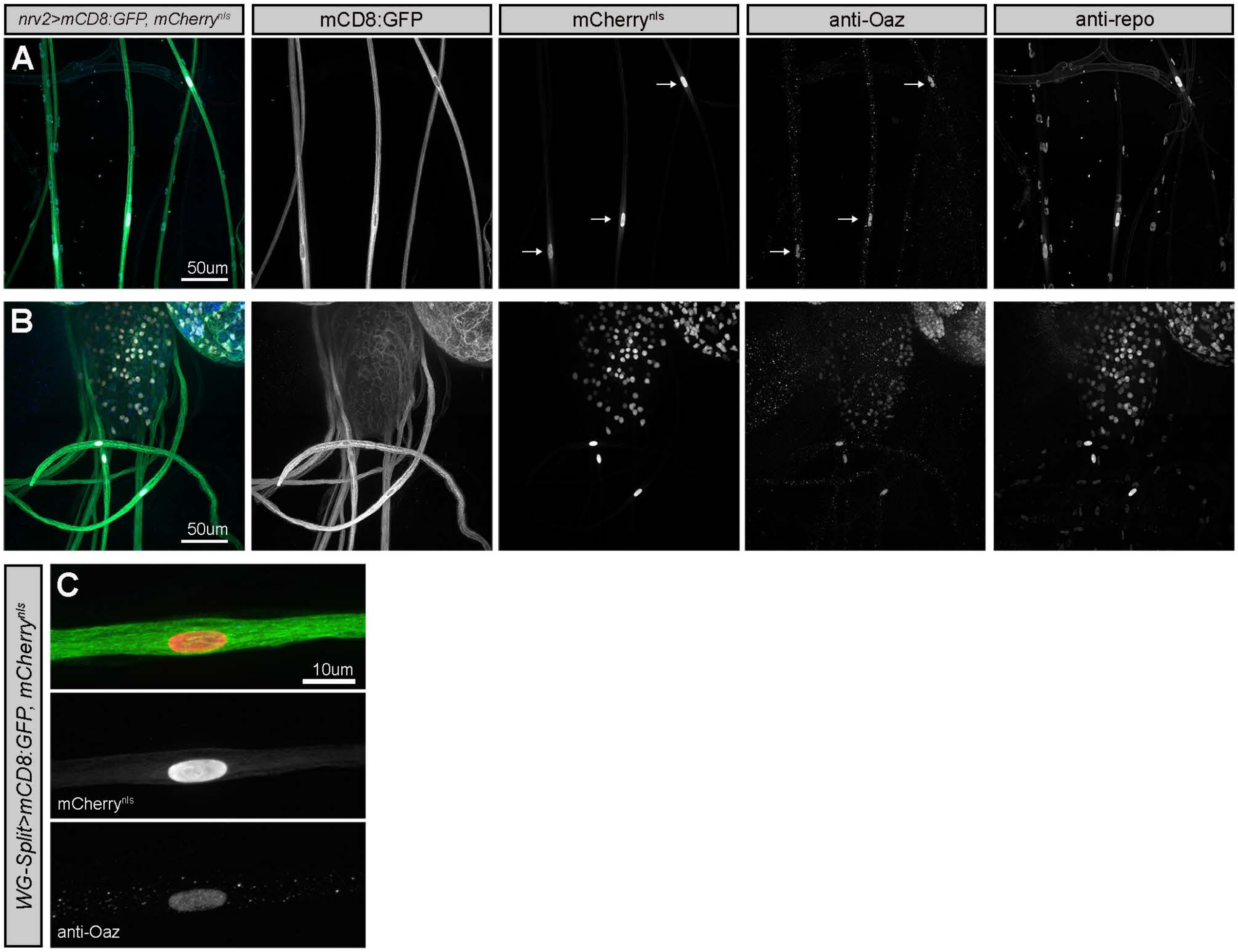

Fig. S2. The transcription actor Oaz labels wrapping glia nuclei along peripheral nerves

(A) *nrv2>mCD8:GFP, mCherry*^{nls} larva stained with anti-Oaz (blue), anti-Repo (cyan). Channels are separated out in subsequent panels. Note only *nrv2>mCherry* positive and not other Repo+ nerve glia express Oaz along nerves (arrows).

(B) CNS and proximal nerves from a *nrv2>mCD8:GFP, mCherry*^{nls} larva, labeled as in (A). Along nerves, only wrapping glia nuclei are labelled with anti-Oaz. Oaz is also expressed in CNS nuclei, including a subset of the glia within the *Nrv2-Gal4* expression pattern and some neurons.

(C) Confocal projection of a single nerve from a *WG-SplitGal4>mCD8:GFP, mCherry*^{nls} larva showing co-localization of anti-Oaz with mCherry.

Development • Supplementary information

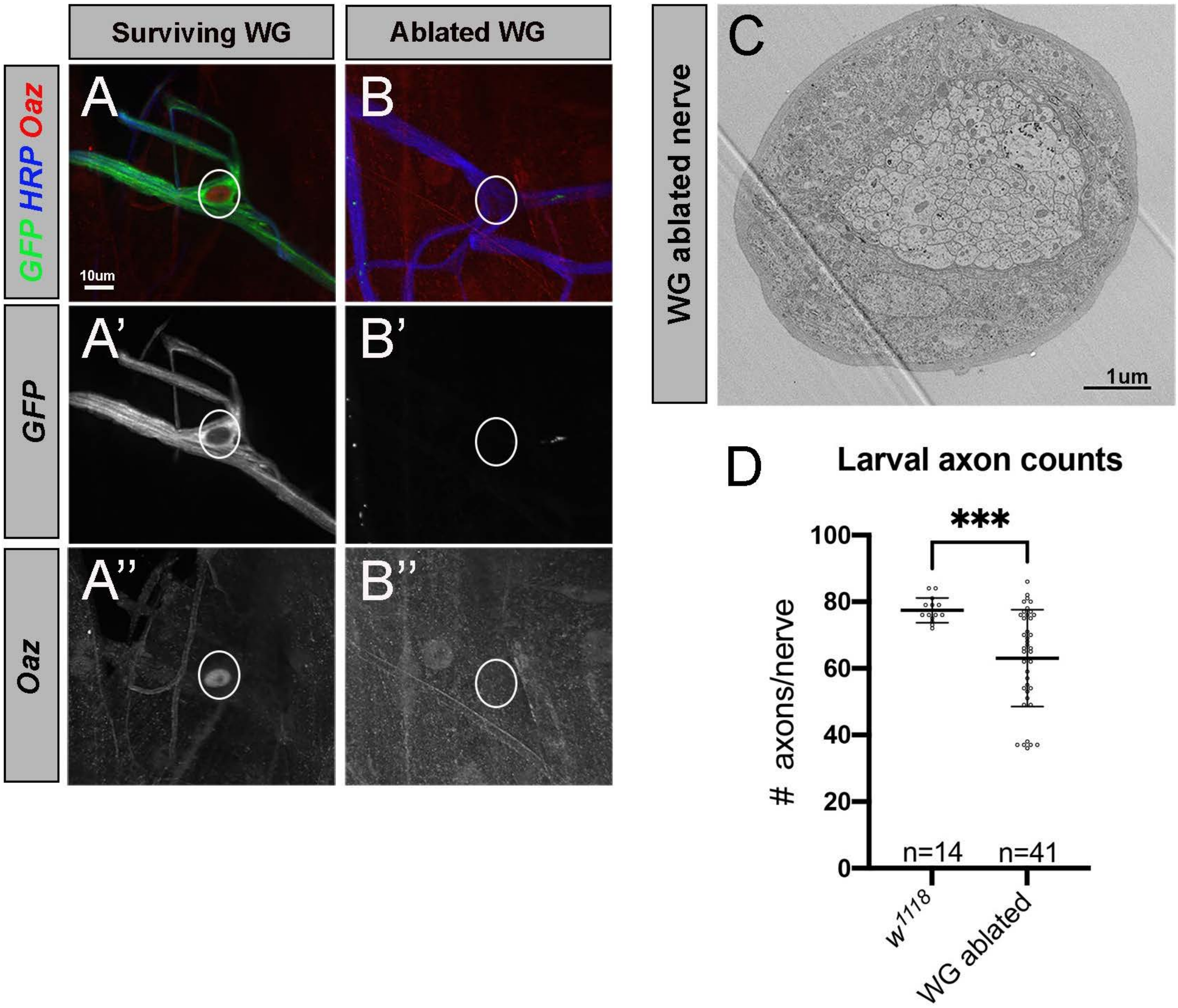

Fig. S3. Ablation veri ication

A subset of larvae used in behavioral testing were subsequently dissected to confirm successful ablations. The absence of GFP (or presence of only small amounts of GFP+ debris) and absence of Oaz staining was observed along nearly all nerves. The only Oaz+ nerve nuclei that were observed were in the few GFP+ wrapping glia found that had escaped ablation.

(A) The cell body region of a surviving ePG5 wrapping glia (von Hilchen et al, 2013) with an Oaz+ nucleus. This surviving cell also continued to express GFP.

(B) The same stereotyped position along the nerve of the ePG5 cell body in an adjacent segment, but there is no GFP nor Oaz staining, confirming the cell has died rather than just down-regulated GFP. **(C)** TEM cross section of an A8/9 nerve from a wrapping glia ablated animal. There is no observable wrapping glia membrane between any axons, but the outer perineurial glia layer appears hypertrophied. About one fifth of nerves from ablated larvae appeared to show this hypertrophy.

(D) Axon counts from TEM analyzed A3-A7 larval nerves. Nerves from WG ablated animals frequently have fewer identifiable axon profiles than the wildtype. *(*w^{1118} average= 77.4 axons; WG-ablated average= 63 axons; Unpaired t-test p=0.0006) n= number of nerves from 4 and 5 larvae, respectively.

Development • Supplementary information

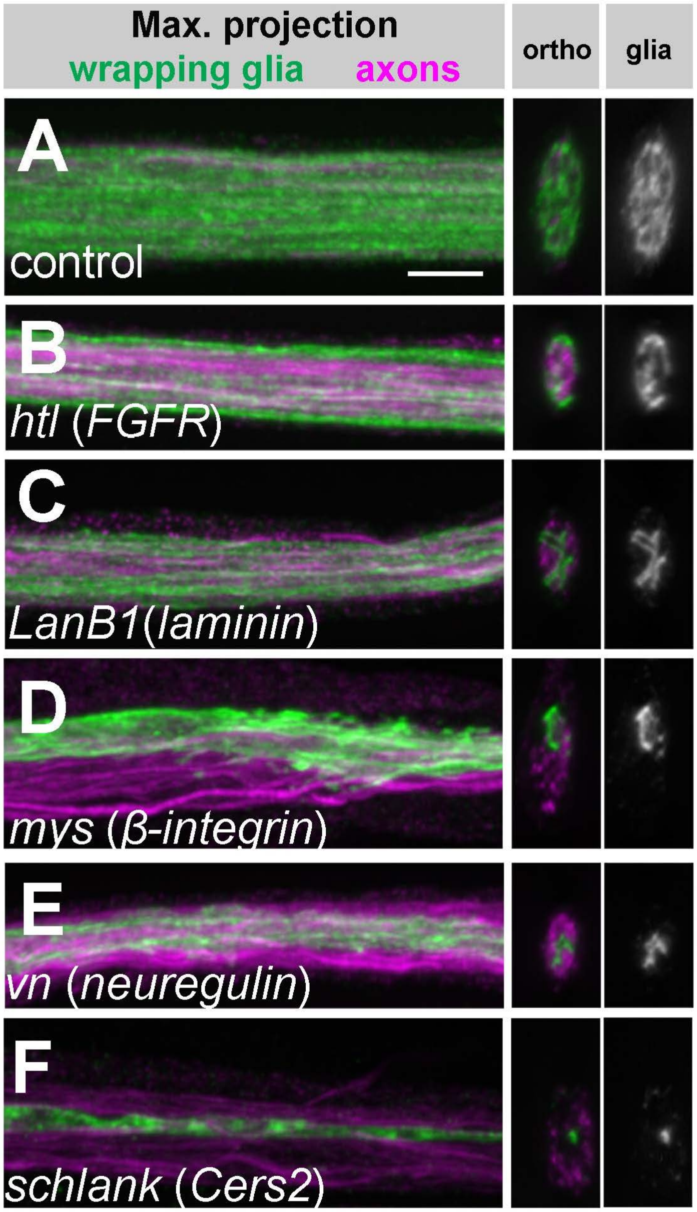

Fig. S4. Genes with conserved roles in glial development uncovered in RNAi screen
(A-F) Wrapping glia (WG) labeled with myr:tdTomato driven by *nrv2-Gal4* (pseudo-colored green). A subset of sensory axons are labeled with anti-Futsch (magenta). Left, maximum confocal projection; right, wrapping glia in cross section. Scale bar 5µm. **(A)** Control. **(B-F)** UAS-RNAi constructs driven in WG. **(B)** *heartless*^{RNAi}. **(C)** *lanB1*^{RNAi}. **(D)** *myospheroid*^{RNAi}. **(E)** *vein*^{RNAi}. **(F)** *schlank*^{RNAi}.

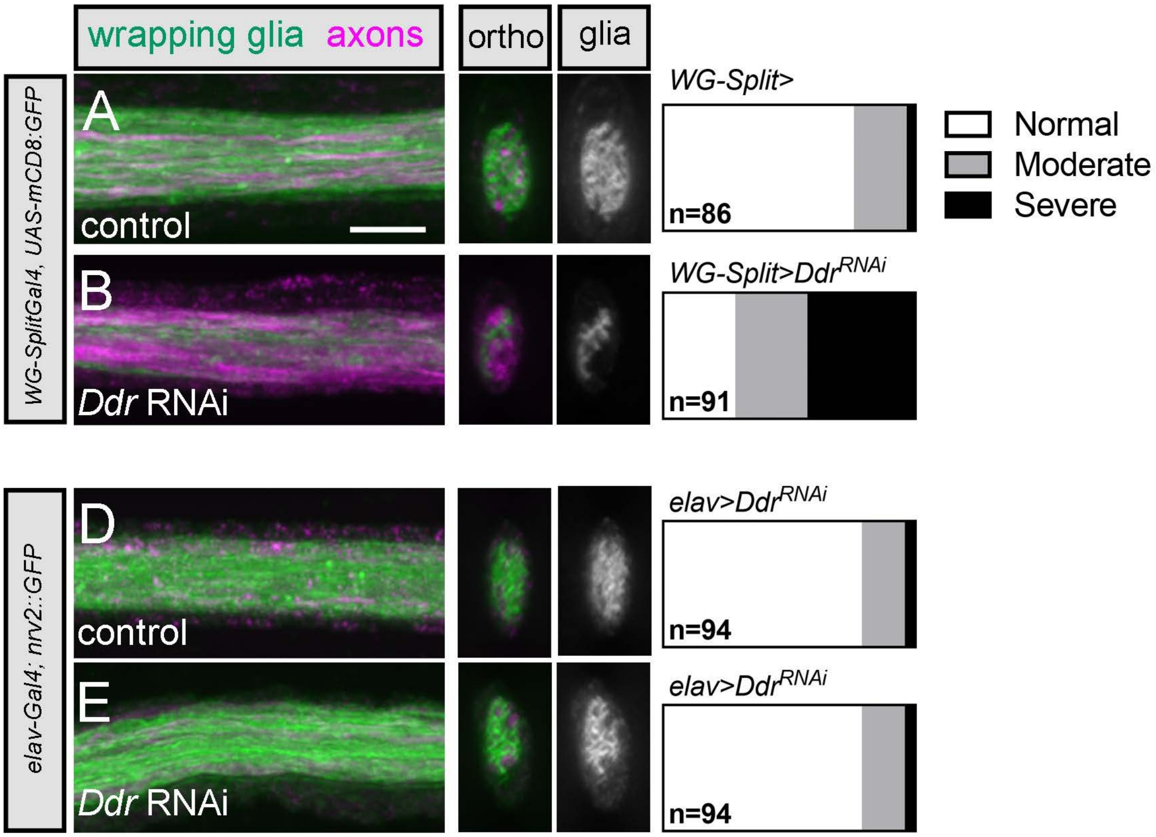

Fig. S5. Cell-type specific knockdown of Ddr with *WG-SplitGal4* and *elav-Gal4*

(A-C) Knockdown of Ddr in wrapping glia using the *WG-SplitGal4* driver disrupts normal wrapping glia morphology, phenocopying *nrv-2-Gal4* knockdown. Wrapping glia visualized with UAS-mCD8:GFP (green); *futsch*+ axons in magenta. **(D-F)** Knockdown of Ddr in neurons using the pan-neuronal driver *elav-Gal4* does not affect wrapping glia morphology. Wrapping glia visualized with nrv2::GFP (green), *futsch*+ axons in magenta. n= # nerves analyzed from 8-13 larvae/condition. Scale bars = 5 µm.

Development • Supplementary information

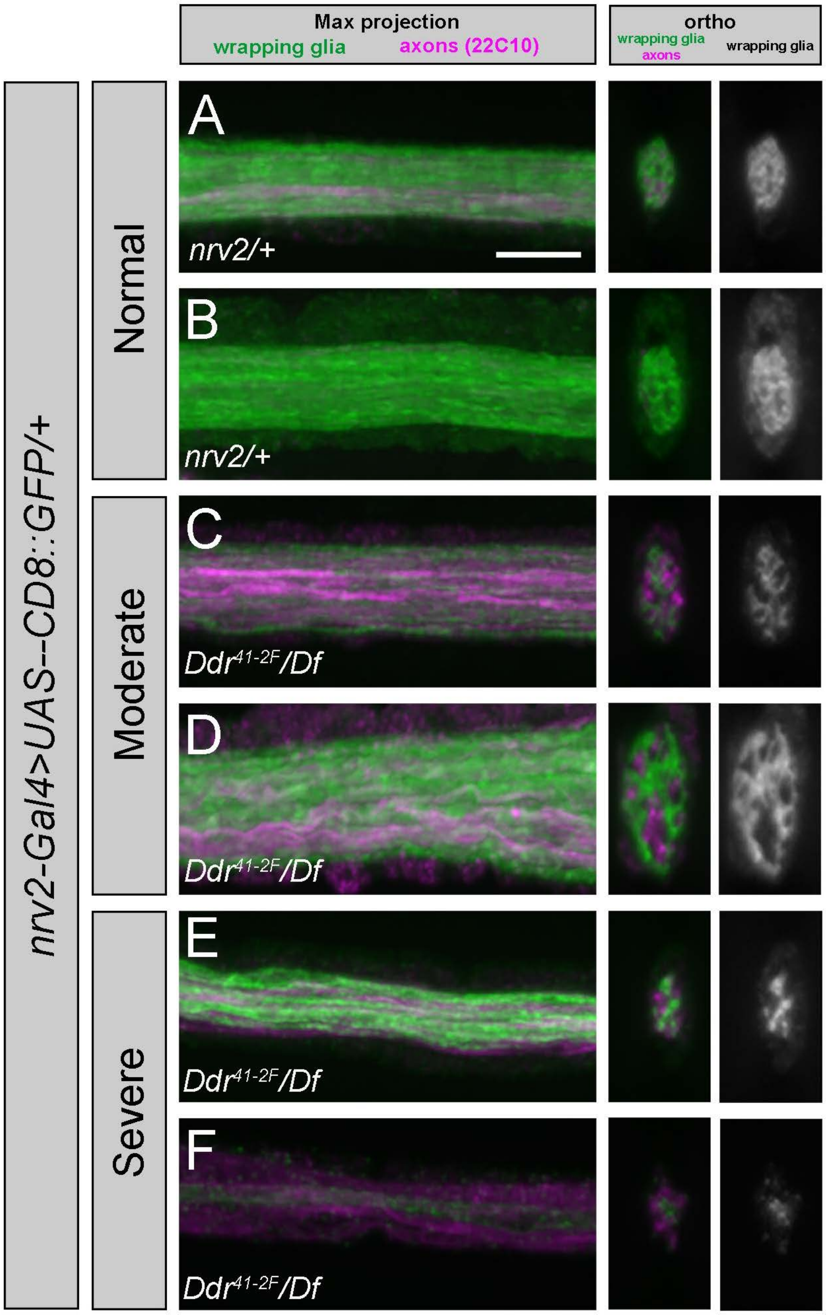

Fig. S6. Categorical scoring of wrapping glia morphology

Representative images of morphologies classified as **(A-B)** "normal" which consists of uniform coverage of the nerve interior with only small coverage gaps. **(C-D)** "moderate" with large gaps/openings in coverage of the nerve cross-section; or **(E-F)** "severe" with minimal wrapping glia processes or even a single glial process visible in cross section. Scale bar = 5 µm

Development • Supplementary information

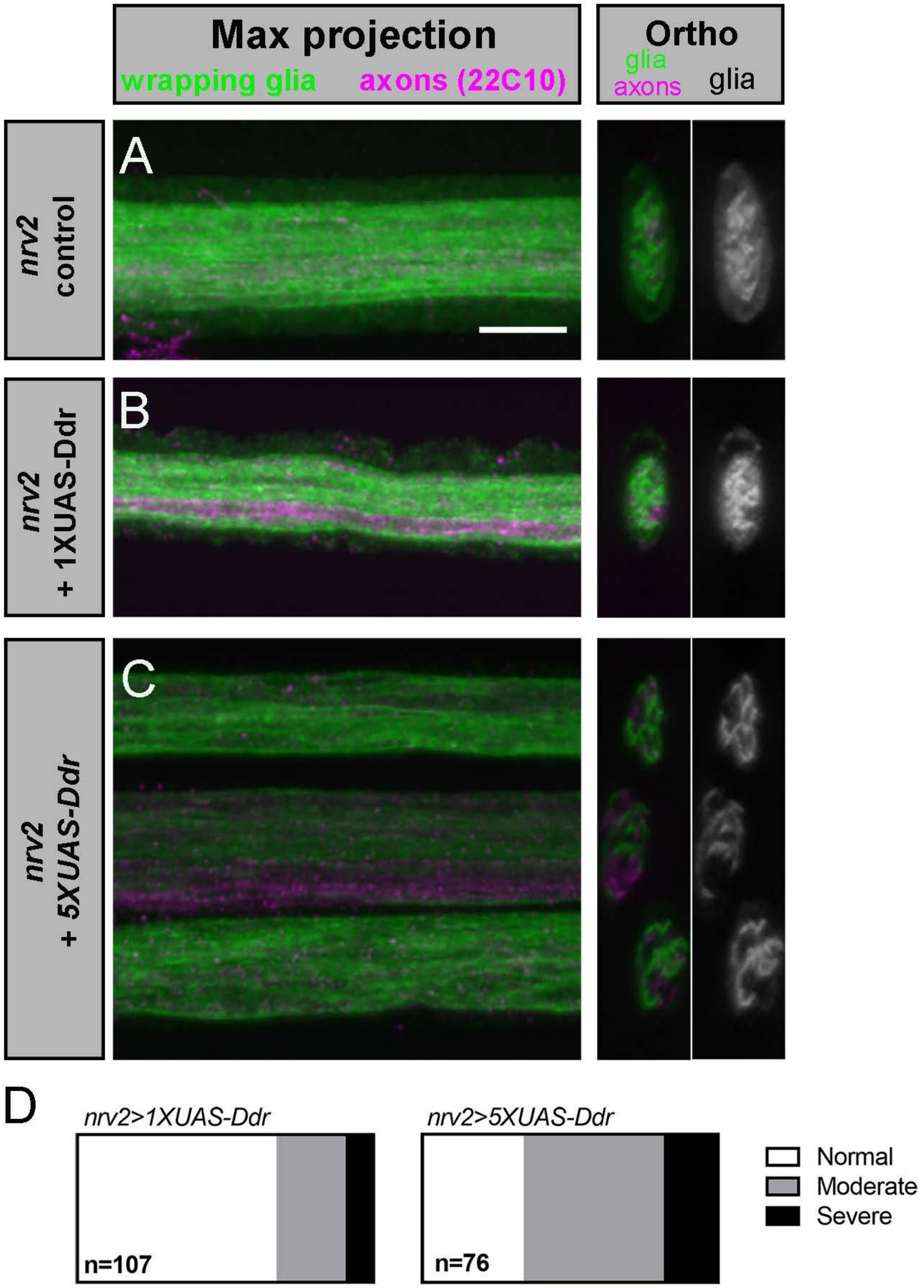

Fig. S7. Overexpression of Ddr using a *5XUAS-Ddr* construct causes morphological abnormalities in control animals

(A) *Nrv2-Gal4* driving *UAS-mCD8:GFP* labels wrapping glia showing normal coverage in orthogonal sections.
(B) Driving *1XUAS-Ddr* in a wildtype background does not alter normal wrapping glia morphology. **(C)** Driving *5XUAS-Ddr* caused abnormal wrapping glia morphology.
(D) Quantification of 1X and 5X constructs in the *nrv2-Gal4* control background. Related to graphs in Figure 2M.
n = # of nerves analyzed from 14 (1XUAS) and 10 (5XUAS) larvae. Scale bar = 5µm

Development • Supplementary information

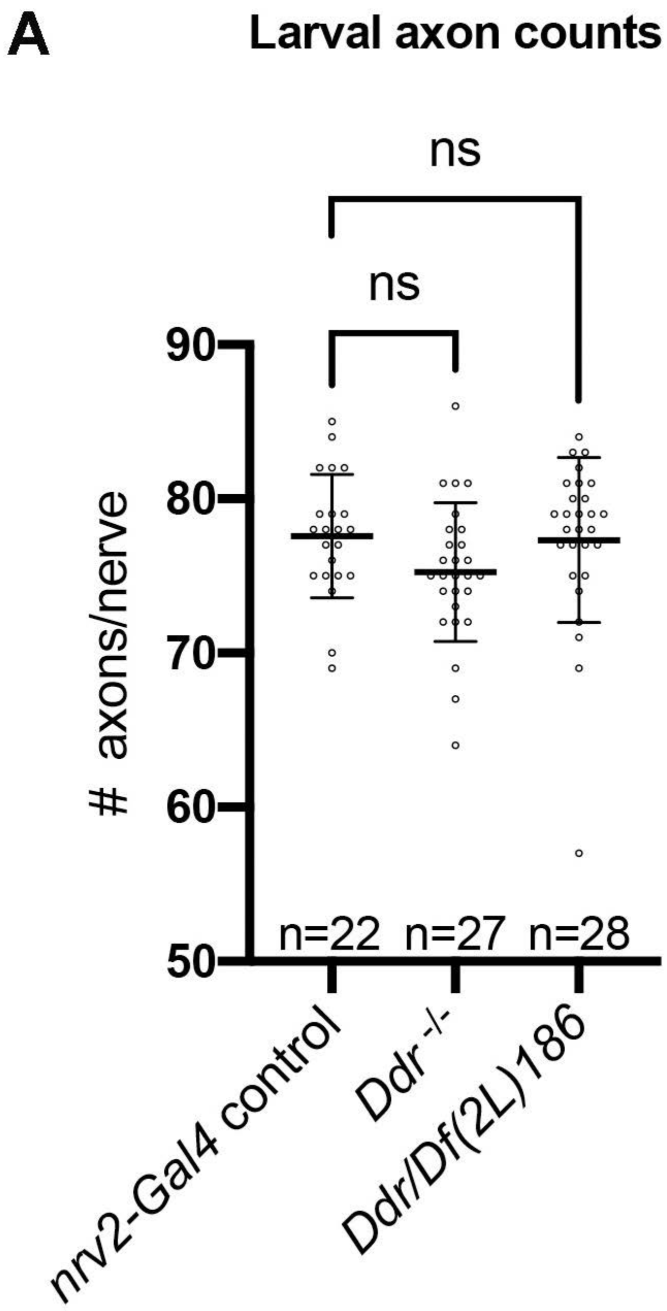

Fig. S8. Loss of Ddr does not affect axon profile number

(A) Axon profiles from A3-A 7 nerves in *nrv2-Gal4/+* (control), *Ddr* mutant, and *Ddr/Df*. One-way AN OVA: *Ddr*$^{-/-}$ vs. control p=0.15; *Ddr/Df* vs. control p=0.97, n= # of nerves.)

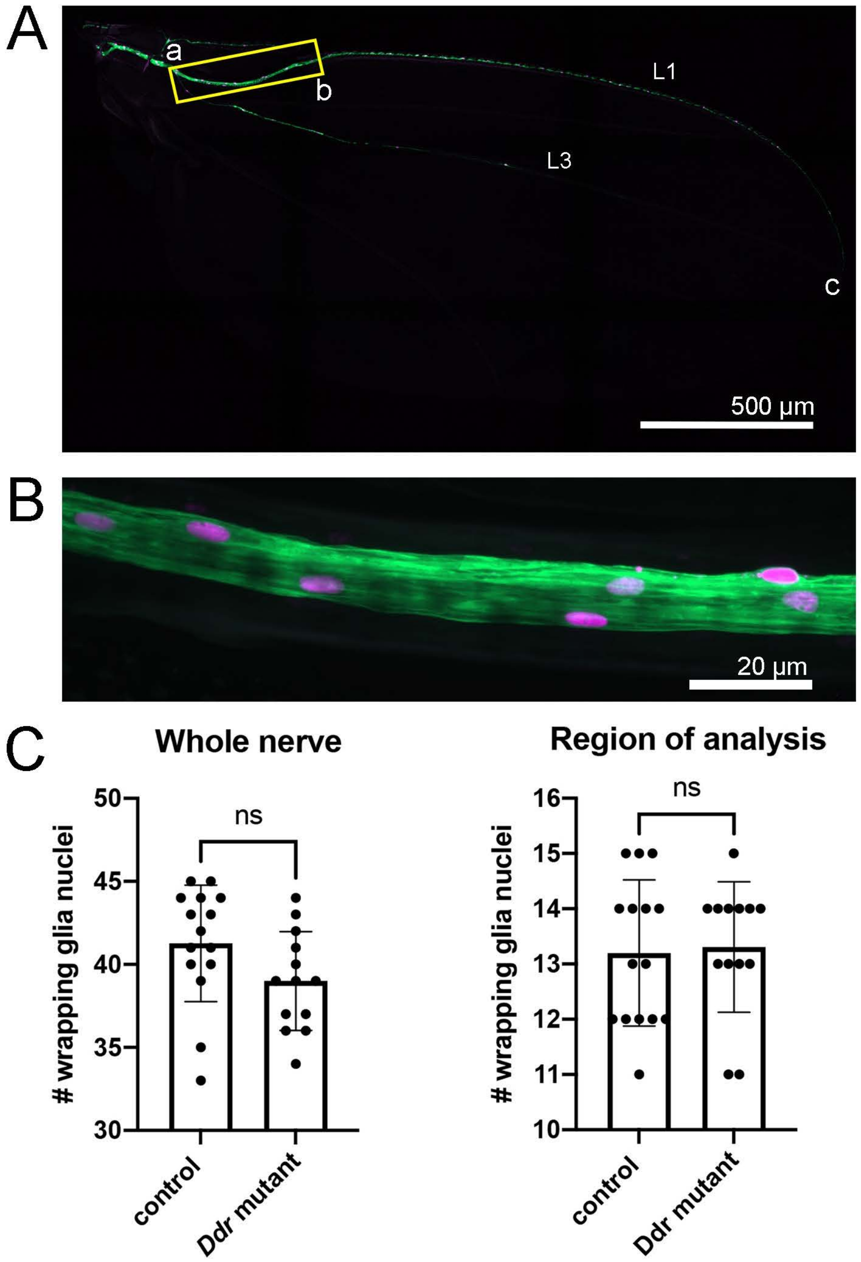

Fig. S9. Loss of Ddr does not affect wrapping glia cell number in the wing
(A) *Nrv-2-Gal4* expression in the wing. Green: wrapping glia membrane, Magenta: wrapping glia nuclei. Cell counts for the whole nerve start at "a", where the L3 vein joins L1 and end at "c" where the nerve ends near the most distal sensory neuron cell body. Wrapping in L1 is analyzed in the area of the yellow box between "a" & "b" the position of the paired campaniform sensilla.
(B) L1 nerve wrapping glia in the region of analysis. Adult nerves have many more wraaping glia as compared to larvae.
(C) Wrapping glia nuclei numbers do not change in *Ddr* mutants, as analyzed at 4 dpe, neither along the entire nerve nor when focusing on the region where ensheathement is analyzed.
Whole neve: 41.27 ± 3.5 vs 39 ± 3.0 nuclei; p= 0.0793, Unpaired t-test
Region of analysis:13.20 ±1.3 vs 13.31± 1.2 nuclei; p= 0.8231, Unpaired t-test
n=15 wings for control, 13 wings for *Ddr*; 1 wing/animal

Development • Supplementary information

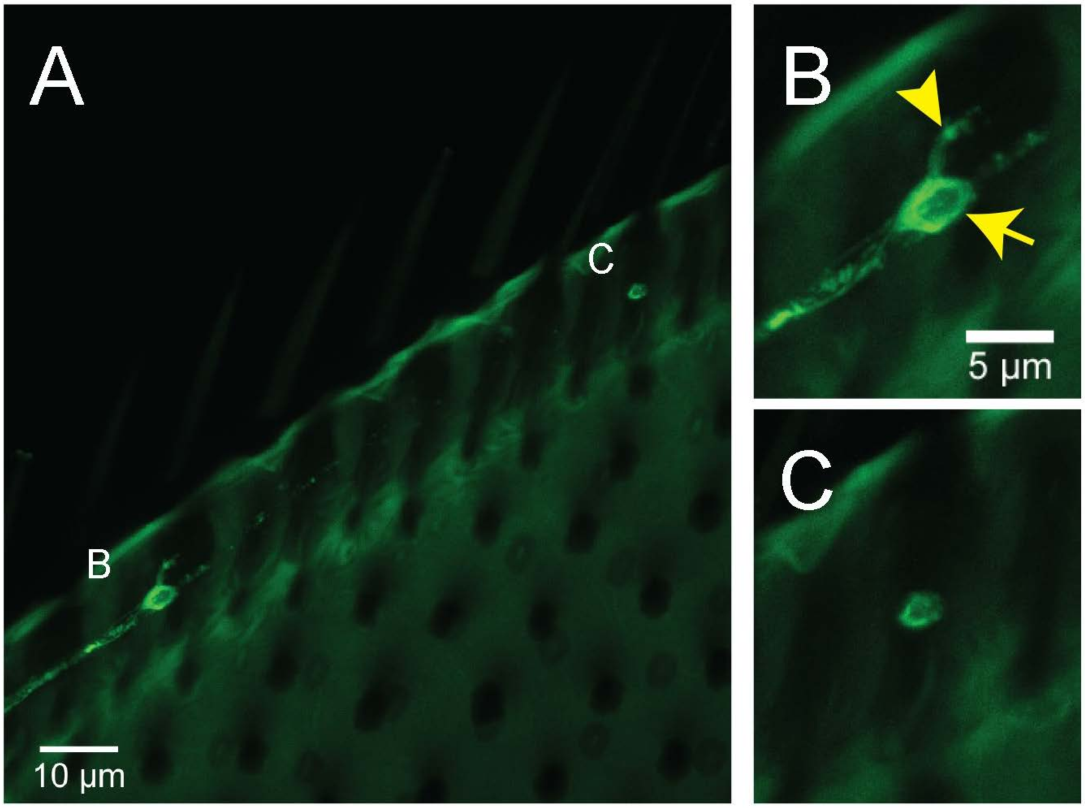

Fig. S10. Healthy vs. dead sensory neuron cell bodies in the wing.
(A) Glutamatergic sensory neurons are positioned along the wing margin. This example depicts two neighboring neurons. One is healthy as evidenced by its intact dendrite (arrowhead) and clear nucleus (arrow) (B). The other is a dead or dying cell body. It lacks a dendrite and has a condensed nucleus and cell body (C).

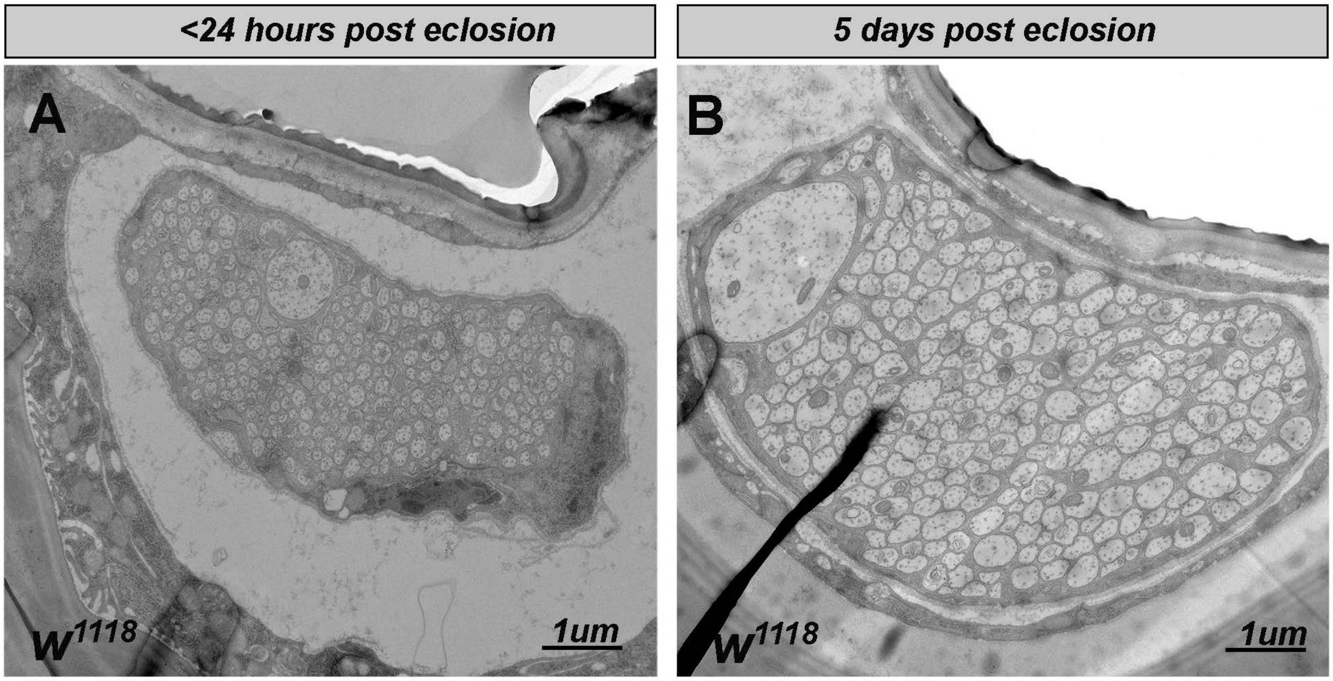

Fig. S11. The dTSM axon increases in caliber between eclosion and 5 dpe

(A) TEM of wild type nerve from a female within 24 hours of eclosion shows that the nerve is overall smaller, as is dTSM immediately after eclosion.

(B) Representative TEM of a wild type wing nerve from a 5 dpe female.

Development • Supplementary information

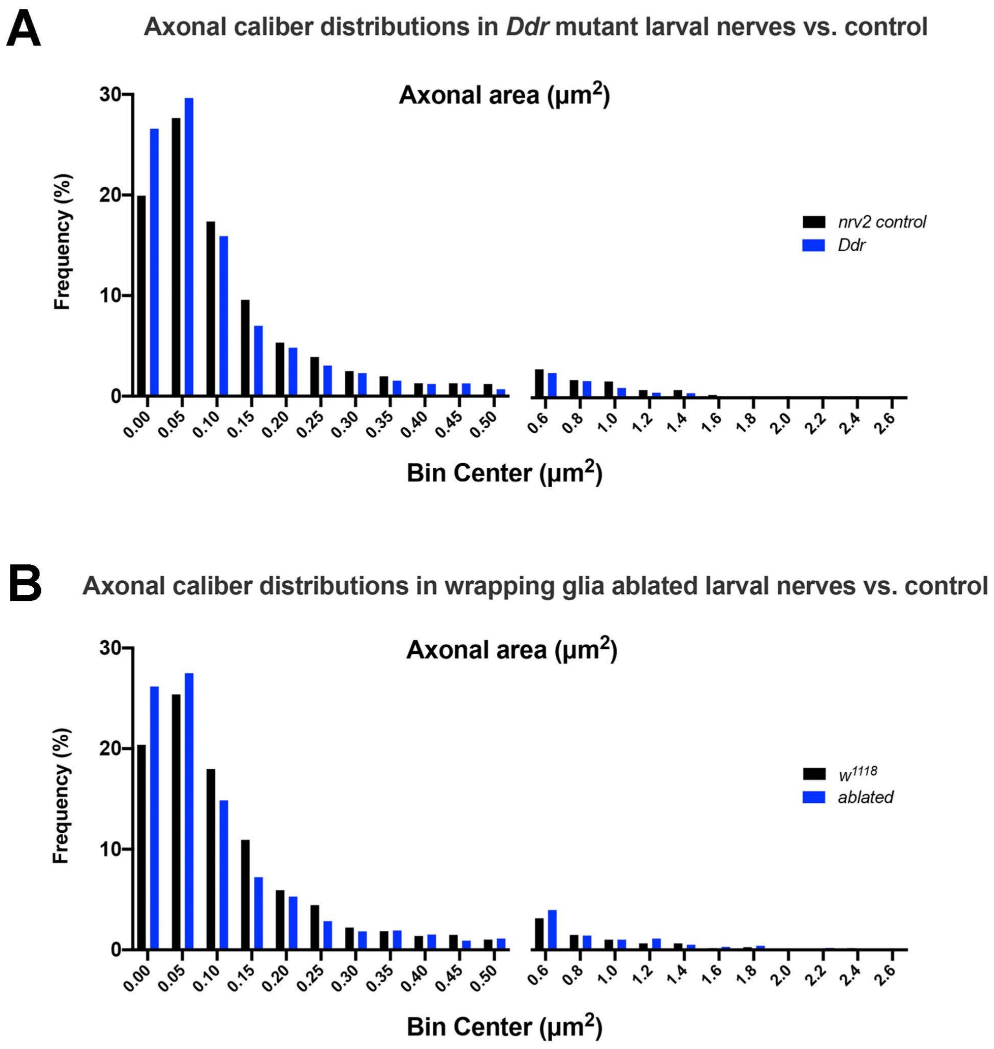

Fig. S12. Axon size distributions in *Ddr* and wrapping glia-ablated larval nerves Frequency distributions of axon caliber (cross-sectional surface area measurements from TEM sections) in 3rd instar nerves. The histograms are heavily skewed towards small axons, note the discontinuous x-axis with different bin sizes for the largest axons.

(A) ***Ddr*** mutant vs. driver control nerves. The distributions are significantly different (Kolmogorov-Smirnov test $p < 0.0001$ with median cross-sectional area: *nrv2* control = 0.085 μm^2 vs. ***Ddr*** = 0.062 μm^2).

(B) Wrapping glia-ablated vs. wild type control frequency distributions. The distributions are significantly different (Kolmogorov-Smirnov test $p < 0.0001$ with median cross-sectional area: control = 0.083 μm^2 and ablated = 0.064 μm^2).

nrv2 control: 18 nerves, 5 larvae, 1410 axons ***Ddr***: 22 nerves, 4 larvae, 1741 axons

w^{1118} control: 14 nerves, 4 larvae, 1079 axons. Ablated: 13 nerves, 5 larvae, 982 axons

Development • Supplementary information

Supplementary Materials and Methods

Fly strains

New fly strains that were generated for this study were constructed as follows:

Ddr$^{41\text{-}2F}$ and *Ddr*$^{13\text{-}1M}$: Deletion alleles of *Ddr* were generated using CRISPR-Cas9 gene editing (Port et al., 2014). Briefly, we selected gRNAs targeting the 3rd and 4th exons of *Ddr* and cloned these sequences into pCFD3d under a U6 promoter. *Ddr_ex3_pCFD3d* and *Ddr_ex4_pCFD3d* plasmids were co-injected into *Drosophila* embryos expressing Cas9 in the germline (BL stock 51324; *w*^{1118}*;; PBac{y[+mDint2]=vas-Cas9 3P3-GFP}*^{VK00027}; Rainbow Transgenics). Progeny of injected flies were screened for large deletions between the target sites in genomic DNA using primers spanning the predicted deletion that would produce a ~360bp product only if a deletion between gRNA sites had occurred. (Without a large deletion a 11.6kB product would be produced.) Animals harboring large deletions were established as individual stocks by crossing to balancer chromosomes and crossing out *vas-Cas9*. Genomic DNA from each stock was sequenced spanning the predicted deletion to determine if any stocks had frameshifts that introduced early stop codons. *Ddr*$^{13\text{-}1M}$ and *Ddr*$^{41\text{-}2F}$ and had such frame shifts which resulted in predicted lengths of 150aa and 134aa respectively. As the shorter allele, *Ddr*$^{41\text{-}2F}$ was selected for further use in experiments. We frequently performed mutant analysis as *Ddr*$^{41\text{-}2F}$ */Df (2L)186* to minimize impact of any unexpected background mutations or suppressors acquired via either the CRISPR strategy or homozygous viable status of the *Ddr*$^{41\text{-}2F}$ allele.

gRNA sequences:
gRNA_Ddr_ex3: GGCCAGTTCGGCCCATG|
ATA**TGG** gRNA_Ddr_ex4: CCTATGTGATTGAGTAC|
TGG**AGG** Deletion detection primers:

ddrEx3_F1: CGGAATTCCACTGCTTTGTT
ddrEx4_R1: CCCAGATGGTTCAGATGGTT

Development • Supplementary information

WG-SplitGal4: Nrv2-Gal4^{DBD} was generated as described in (Coutinho-Budd et al., 2017). *IT.0117-Gal4* was converted to *IT.0117-VP16AD* with a series of genetic crosses as described in Gohl et al, 2011. Once *IT.0117-VP16AD* was established as a stock it was recombined with *Nrv2-Gal4^{DBD}* to generate a stable *WG-SplitGal4* stock on chromosome III.

5XUAS-Ddr and *1XUAS-Ddr:* A full length 3.165kb Ddr cDNA based on the Ddr-PG isoform sequence listed on Flybase was synthesized and cloned into pUC19 by Genscript USA (Pistacaway, NJ 08854 USA). To construct 5XUAS-Ddr, Ddr was amplified from pUC19-Ddr using primers to introduce a 5' BglII site and Kozak sequence and 3' XhoI site. BglII and XhoI were then used to clone Ddr into the pattB-5xUAS vector. To construct 1xUAS-Ddr, the Ddr sequence was isolated from pattB-5xUAS-Ddr using BglII/XhoI and cloned into the pattB-1xUAS vector. The constructs were injected into y^{1} w^{67c23}; P{CaryP}attP154 embryos (Best Gene) for site-directed intregation onto the 3rd chromosome at position 97D2. Transformants were identified by eye color and stocks were established from single male founders by crossing to balancer females.

Cloning primers:

Forward: GAATACAAGAAGAGAACTCTGAATagatctcaaaacATGCCTGCAATAAAGTTACAAGAATCG

Reverse: gctagcatggtaccataggcctatctcgagTCAATACATGTGTGTATGGGTCTG

Generation of the anti-Oaz antibody

Oaz was identified as a potential wrapping glia marker in an enhancer trap screen where it labeled only three nuclei per abdominal nerve (M. Freeman, unpublished). Rabbits were immunized with a purified fusion peptide corresponding to the C-terminal 123 amino acids of both predicted Oaz isoforms. The fusion peptide was generated by cloning the corresponding cDNA fragment from clone AT08673 (Berkeley Drosophila Genome Project) into pET28a and

Development • Supplementary information

inducing protein expression in bacteria. The resulting serum produces optimal staining if a stock of 1:50 is pre-absorbed against either fixed *Drosophila* embryos or fixed 3rd instar larval carcasses overnight at 4°C. This preabsorbed sera can then be diluted 1:100 for a final concentration of 1:5000. Oaz labels nuclei in the CNS (subsets of glia and neurons) and muscles in addition to wrapping glia nuclei along the peripheral nerves. To confirm that the 3 nuclei labeled by Oaz along the nerves correspond to wrapping glia, we used wrapping glia driver lines to drive nuclear reporter expression and check for co-localization with anti-Oaz, as shown in Fig. S2. These experiments confirm that along the nerves, only wrapping glia nuclei are labeled and can thus be positively identified by anti-Oaz staining.

Oaz peptide sequence for immunization:
NHMGEHGAHSRPYDCNLCPEKFFFRAELEHHQRGHELRPQARPPAAKVEVPSIRNTSPGQSPVRSPTIVKQE
LYETDTVESAGVEDEPENHPDEEEYIEVEQMPHETRPSGIGSQLERSTSSA

Electron microscopy

Larvae: 3rd instar larvae were dissected as fillets in ice cold 0.1M cacodylate buffer pH 7.4 (EMS). The buffer was immediately substituted for 2.5% glutaraldehyde in 0.1M cacodylate buffer and left to fix with gentle agitation for 30 minutes at RT, after which dissecting pins were removed and fillets transferred to fresh fixative overnight at 4°C. After 3x 10 minute washes with gentle agitation in 0.1M cacodylate buffer, fillets were incubated in 1% OsO4 (EMS) prepared in ddH2O for 1 hour at RT. After 3x 10 minute washes in ddH2O, fillets were taken through a 4-dilution EtOH dehydration series, with 30%, 50%, 70%, and 95% EtOH for 10 minutes each followed by 2x 10 minute incubation in 100% EtOH, and 2x 10 minute incubation with propylene oxide (PO). Infiltration with 1:1 PO:Epon-812 resin proceeded overnight at RT on a rotating shaker followed by 2 hours infiltration with 100% Epon-812. Larval fillets were flat embedded between 2 sheets of Aclar plastic (EMS) and cured overnight at 60C before being

Development • Supplementary information

trimmed and re-embedded in coffin molds to prepare for sectioning. 70nm sections were collected from ~200um posterior to the tip of the VNC and placed on 200 mesh copper grids (EMS). Grids were post-stained with 5% uranyl acetate for 20 minutes and Reynolds lead citrate for 8 minutes before being imaged on a Technai T12 electron microscope at 80kV equipped with and AMT digital camera and software.

Wings: To facilitate fixation of the nerve within the wing cuticle we used microwave assisted fixation. Our protocol was adapted from protocols for zebrafish larvae (Cunningham and Monk, 2018; Czopka and Lyons, 2011). Adult flies were anesthetized on CO2 pads and wings were removed with fine dissection scissors and forceps taking care not to touch or crush the anterior edge of the wing. Wings were immediately placed in Eppendorf tubes with modified Karnofsky's fixative (2% glutaraldehyde, 4% paraformaldehyde in 0.1M sodium cacodylate buffer, pH 7.4; EMS). Wings from the same genotype were collected in each tube for primary fixation in a Pelco Biowave microwave (Ted Pella) with the settings: 100W for 1 min, OFF for 1 min x2; 450W for 20 seconds, OFF for 20 seconds x5. Wings were kept in the fixative overnight at 4°C before proceeding. Following 3x10 minute washes with fresh 0.1M sodium cacodylate buffer, 2% OsO4 was added to each tube and samples were again microwaved at 100W for 1 min, OFF for 1 min x2 followed by 450W for 20 seconds, OFF for 20 seconds, x5. OsO4 is then washed out with 3x10minutes washes with ddH20. In bloc UA staining is then performed by adding 8% UA to each tube and microwaving (450W for 1 min, OFF for 1 min, 450W for 1 min). The UA is washed out with 3x10 minute washes with ddH20 and then the dehydration series begins. Samples are taken through an EtOH dilution series of 30%, 50%, 70%, 80%, 95%, each followed by a microwave cycle (250W for 45 seconds) prior to proceeding to the next step. Three changes of 100% EtOH, each followed by a microwave cycle (250W for 1 min, OFF for 1

Development • Supplementary information

min, 250W for 1 min) is followed by 3 changes of 100% acetone with the same microwave settings. The final acetone wash is used to move samples to glass dram vials. The pure acetone is replaced with a 1:1 mixture of acetone and Embed 812 (EMS) and allowed to infiltrate samples overnight on a rotating shaker. All microwave steps are carried out with samples in a chilled circulating water bath to keep sample temperature below 20°. Great care must be used when switching solutions as the wings are very hydrophobic and will not sink in liquids until the 80% EtOH step and can be easily lost.

The next day, the 1:1 acetone:Embed-812 mixture is replaced with 100% fresh Embed-812 resin and allowed to infiltrate for at least 1 hour on a rotating shaker. Wings are then flat embedded between Aclar sheets and cured overnight in a 60°C oven. Embedded wings are imaged on Olympus upright microscope to check for any signs of L1 nerve damage. Wings that have tears or scars along the L1 vein are not selected for sectioning and analysis. Intact flat embedded wings are then trimmed close to the ROI with a warm razor blade and re-embedded in coffin molds to facilitate sectioning. 70nm sections are collected on 100mesh formvar film coated grids and counterstained as above with 5% uranyl acetate for 20 minutes and Reynolds lead citrate for 8 minutes before being imaged on a Tecnai T12 electron microscope at 80kV or 120kV equipped with and AMT digital camera and software.

Table S1. Fly strains

Stock	Source	Reference
w^{1118}		
nrv2-gal4 on 2nd	Bloomington (BL) #6800	(Sun et al., 1999)
nrv2-gal4 on 3rd	BL #6799	(Sun et al., 1999)
repo-Gal4	BL #7415	(Sepp et al., 2001)
nrv2::GFP	gift of C. Klambt lab	(Stork et al., 2008)
VGlut-QF2	BL #60315	(Diao et al., 2015)
OK371(VGlut)-QF2	BL #66473	(Lin and Potter, 2016)
UAS-reaper (II)	BL #5824	(Aplin and Kaufman, 1997)
IT.0117-Gal4	BL #62647	(Gohl et al., 2011)
$IT.0117^{VP16AD}$	this study	
$nrv2\text{-}Gal4^{DBD}$	generated in Freeman Lab	(Coutinho-Budd et al., 2017)
Mp^{f07253}	BL #19062	
$Ddr^{41\text{-}2F}$	this study	
$Ddr^{13\text{-}1M}$	this study	
Df(2L)BSC186	BL #9614	(Cook et al., 2012)
1xUAS-Ddr	this study	
5xUAS-Ddr	this study	
$CH321\text{-}94A23^{VK31}$ (Ddr BAC)	Genetivision #P3-25	(Venken et al., 2009)
UAS-CD8::GFP on 2nd	BL #108068	(Lee and Luo, 1999)
UAS-CD8::GFP on 3rd	BL #5130	(Lee and Luo, 1999)
10xUAS-myr:tdTomato	BL #32222	(Pfeiffer et al., 2010)
UAS-mCherrynls	BL #38424	
UAS-myrGFP.v5-P2A-H2BmCherry.HA	gift from J. Dubnau Lab	(Chang et al., 2019)
10xQUAS-6XGFP	BL #52264	
$Mp^{MI09316\text{-}GFSTF.0}$	BL #60567	
WG-SplitGal4	this study	
UAS-htlRNAi	VDRC #27180	
UAS-LanB1 RNAi	VDRC #23119	
UAS-mys-RNAi	VDRC #103704	
UAS-vnRNAi	VDRC #109437	
UAS-schlankRNAi	VDRC #109418	
UAS-Mp RNAi	VDRC #38188	
UAS-Ddr-RNAi #1	VDRC #29720	
UAS-Ddr-RNAi #2	VDRC #51719	
nsyb-Gal4	BL #51635	
$elav\text{-}Gal4^{C155}$	BL #458	

Table S2. Complete genotypes of animals used in each figure

Click here to download Table S2

References

Aplin, A. C. and Kaufman, T. C. (1997). Homeotic transformation of legs to mouthparts by proboscipedia expression in Drosophila imaginal discs. *Mech Develop* 62, 51–60.

Chang, Y.-H., Keegan, R. M., Prazak, L. and Dubnau, J. (2019). Cellular labeling of endogenous retrovirus replication (CLEVR) reveals de novo insertions of the gypsy retrotransposable element in cell culture and in both neurons and glial cells of aging fruit flies. *Plos Biol* 17, e3000278.

Cook, R. K., Christensen, S. J., Deal, J. A., Coburn, R. A., Deal, M. E., Gresens, J. M., Kaufman, T. C. and Cook, K. R. (2012). The generation of chromosomal deletions to provide extensive coverage and subdivision of the Drosophila melanogaster genome. *Genome Biol* 13, R21.

Coutinho-Budd, J. C., Sheehan, A. E. and Freeman, M. R. (2017). The secreted neurotrophin Spätzle 3 promotes glial morphogenesis and supports neuronal survival and function. *Gene Dev* 31, 2023–2038.

Cunningham, R. L. and Monk, K. R. (2018). Schwann Cells, Methods and Protocols. 385–400.

Czopka, T. and Lyons, D. A. (2011). Chapter 2 Dissecting Mechanisms of Myelinated Axon Formation Using Zebrafish. *Methods Cell Biol* 105, 25–62.

Diao, F., Ironfield, H., Luan, H., Diao, F., Shropshire, W. C., Ewer, J., Marr, E., Potter, C. J., Landgraf, M. and White, B. H. (2015). Plug-and-play genetic access to drosophila cell types using exchangeable exon cassettes. *Cell reports* 10, 1410–1421.

Gohl, D. M., Silies, M. A., Gao, X. J., Bhalerao, S., Luongo, F. J., Lin, C.-C., Potter, C. J. and Clandinin, T. R. (2011). A versatile in vivo system for directed dissection of gene expression patterns. *Nature methods* 8, 231–237.

Lee, T. and Luo, L. (1999). Mosaic analysis with a repressible cell marker for studies of gene function in neuronal morphogenesis. *Neuron* 22, 451–461.

Lin, C.-C. and Potter, C. J. (2016). Editing Transgenic DNA Components by Inducible Gene Replacement in Drosophila melanogaster. *Genetics* 203, 1613–1628.

Pfeiffer, B. D., Ngo, T.-T. B., Hibbard, K. L., Murphy, C., Jenett, A., Truman, J. W. and Rubin, G. M. (2010). Refinement of tools for targeted gene expression in Drosophila. *Genetics* 186, 735–755.

Port, F., Chen, H.-M., Lee, T. and Bullock, S. L. (2014). Optimized CRISPR/Cas tools for efficient germline and somatic genome engineering in Drosophila. *Proc National Acad Sci* 111, E2967–E2976.

Sepp, K. J., Schulte, J. and Auld, V. J. (2001). Peripheral glia direct axon guidance across the CNS/PNS transition zone. *Developmental biology* 238, 47–63.

Stork, T., Engelen, D., Krudewig, A., Silies, M., Bainton, R. J. and Klämbt, C. (2008). Organization and function of the blood-brain barrier in Drosophila. *The Journal of neuroscience : the official journal of the Society for Neuroscience* 28, 587–597.

Sun, B., Xu, P. and Salvaterra, P. M. (1999). Dynamic visualization of nervous system in live Drosophila. *Proceedings of the National Academy of Sciences of the United States of America* 96, 10438–10443.

Venken, K. J. T., Carlson, J. W., Schulze, K. L., Pan, H., He, Y., Spokony, R., Wan, K. H., Koriabine, M., Jong, P. J. de, White, K. P., et al. (2009). Versatile P[acman] BAC libraries for transgenesis studies in Drosophila melanogaster. *Nat Methods* 6, 431–434.
