# Supplementary material for: Discoidin domain receptor regulates ensheathment, survival and caliber of peripheral axons
Source: Development. 2022 Dec 13;149(23):dev200636. doi: 10.1242/dev.200636 (PMC10112903; doi:10.1242/dev.200636)
Supplement: Supplementary file 1 [file develop-149-200636_review_history.pdf]

Discoidin domain receptor regulates ensheathment, survival and caliber of peripheral axons

Megan M. Corty, Alexandria L. Hulegaard, Jo Q. Hill, Amy E. Sheehan, Sue A. Aicher and Marc R. Freeman
DOI: 10.1242/dev.200636

Editor: James Briscoe

Review timeline

Original submission:	24 February 2022
Editorial decision:	28 March 2022
First revision received:	5 October 2022
Accepted:	27 October 2022

Original submission

First decision letter

MS ID#: DEVELOP/2022/200636

MS TITLE: Discoidin domain receptor regulates ensheathment, survival, and caliber of peripheral axons

AUTHORS: Megan M Corty, Alexandria P Lassetter, Jo Q. Hill, Amy E Sheehan, Sue A Aicher, and Marc R Freeman

I have now received all the referees' reports on the above manuscript, and have reached a decision. The referees' comments are appended below, or you can access them online: please go to BenchPress and click on the 'Manuscripts with Decisions' queue in the Author Area.

As you will see, the referees express considerable interest in your work, but have some significant criticisms and recommend a substantial revision of your manuscript before we can consider publication. I would encourage you to address the issues raised by the referees, most of which involve clarifications or providing additional information. I will be happy to receive a revised version of the manuscript. Your revised paper will be re-reviewed by one or more of the original referees, and acceptance of your manuscript will depend on your addressing satisfactorily the reviewers' major concerns. Please also note that Development will normally permit only one round of major revision. If it would be helpful, you are welcome to contact us to discuss your revision in greater detail. Please send us a point-by-point response indicating your plans for addressing the referee's comments, and we will look over this and provide further guidance.

Please attend to all of the reviewers' comments and ensure that you clearly highlight all changes made in the revised manuscript. Please avoid using 'Tracked changes' in Word files as these are lost in PDF conversion. I should be grateful if you would also provide a point-by-point response detailing how you have dealt with the points raised by the reviewers in the 'Response to Reviewers' box. If you do not agree with any of their criticisms or suggestions please explain clearly why this is so.

© 2022. Published by The Company of Biologists under the terms of the Creative Commons Attribution License (https://creativecommons.org/licenses/by/4.0/).

Reviewer 1

Advance summary and potential significance to field

"Discoidin domain receptor regulates ensheathment, survival, and caliber of peripheral axons" by Corty et al reports a set of experiments employing Drosophila as a model, which have uncovered several novel roles for both wrapping glia and the Discoidin domain receptor in regulating peripheral nerve formation and maintenance. The first set of data in the manuscript focus on the analysis of the consequences of specifically ablating peripheral wrapping glia (through a clever gene targeting approach), finding that this disrupts both axonal integrity and larval fly behaviour. From there, the manuscript describes a series of studies that aimed to better understand the mechanisms of nerve wrapping, particularly given that the multi-axon wrapping seen in larval Drosophila nerves, analogous to that which is mediated by Remak Schwann cells in vertebrates, is relatively understudied. A powerful wrapping glia-driven RNAi-based screen identified several factors that disrupted the morphology of wrapping glia, of which Ddr was one, and the principal focus on this manuscript. The identificaiton of ddr as a regulator of wrapping glia morphology was corroborated by the generation of mutant lines through CRISPR-mediated gene targeting. In addition to characterising phenotypes in both constitutive and wrapping glia specific ddr mutants in the larva and adult fly, the study also identifies a Drosophila Collagen as a likely ligand for ddr in mediating wrapping glial cell morphology in the larval stage. There is an abundance of important and novel information in this manuscript and the conclusions drawn are generally very well supported. Future studies will determine whether the function of ddr will be conserved in the vertebrate nervous system.

Comments for the author

While some may consider testing conservation of ddr function in a vertebrate an important question to address experimentally up front, I personally think that the study is of a sufficient enough advance to warrant publication as a standalone Drosophila-focussed piece and for vertebrate conservation to be tackled separately. This said, I do, however, have a number of points that I think need to be addressed before publication, some of which are suggestions for extending the analyses a bit further, and some of which will be requests for clarification based on my lack of direct expertise in fly genetics.
1. Regarding wrapping glia ablation, can the authors please measure axon diameter/ caliber in animals without wrapping glia, given that the TEM data is to hand. This would help better match up and understand the similarities and differences in phenotypes between larvae and adults (discussed further below).
2. Related to this point, is it possible to ablate wrapping glia in the adult, using the new split gal4 model, and if so, can the effect on diameter be assessed. I wouldn't consider this an essential request, but rather a potential extension, depending on feasability.
3. I am unclear, not being a fly expert, whether the wrapping glia of larvae are the same cells in the adult. It appears that the mechanisms of wrapping are quite distinct, with multi-axon wrapping prominent in larvae, and single axon wrapping prominent in the adult. Do the same cells move to wrapping fewer axons, how does cell number increase over time, or is the population turned over. To me, the most unclear aspect of the study was the disconnect between larval and adult phenotypes. I think that non-experts would benefit greatly from knowing how wrapping glia of larvae and adults were related. Similarly, I thinkit would be important to actually quantify wrapping glial cell number in larvae and adults. Is the number reduced in larvae, or simply the morphology? If also number, does this catch up in adult, explaining lack of obvious wrapping phenotype.
4. Again, related to larval to adult analyses, I'm afraid I wasn't able to tell if the cell-type sepific driver (repo) used to disrupt ddr in adults were such that larval ddr function was intact and ddr was only disrupted in adult wrapping glia. This could simply be stated for clarity and would add strength to interpretation of adult-specific phenotypes (or lack thereof). One point that is not clear in this regard is to what extent phenotypes have transitioned from being wrapping-glia specific to broader glial specific. Are any of the adult phenotypes truly wrapping glia specific? Much as per the question regarding adult wrapping glia ablation, I don't know whether it is possible to do wrapping glia specific targeting of ddr through to adult stages. I just fear that naive readers might conflate wrapping glia functions in larvae with glial functions in adults. It would be important to pre-empt this confusion/ conflation.

© 2022. Published by The Company of Biologists under the terms of the Creative Commons Attribution License (https://creativecommons.org/licenses/by/4.0/).

Minor point for discussion. There is a disconnect in the behavioural consequences of wrapping glia ablation and ddr loss of function, which is discussed. However, coudl it also be that the effect of the wrapping glia ablation on behaviour is due to the ablation aspect, i.e. the induciton of cell death and associated inflammatory response? This could be discussed, as it is broadly relevant to studies that ablate cells and assess behaviour: how do such manipulations reflect essential function of the cell type versus the inevitable parallel consequences of cell death.

Reviewer 2

Advance summary and potential significance to field

In this study, Corty et al. set out to dissect the molecular mechanisms that govern wrapping glial ensheathment of peripheral nerves, a process which is incompletely understood.
Focusing on the larval abdominal neurons, the authors used an RNAi screen against cell-surface and secreted proteins in wrapping glia and identified a new molecular player: the Discoidin Domain Receptor (Ddr) as a regulator of glial ensheathment in larval nerves. Overall, while the manuscript is well-written and clear, the authors could do more to better place the role of Ddr in context of other known glial wrapping regulators and advance our understanding of the process beyond merely adding a new player. In addition several points need to be addressed to support some of the interpretations of the manuscript.

Comments for the author

1. To demonstrate that wrapping glia are essential to support neural circuit function, the authors genetically ablated wrapping glia. As a result, they observed a significant reduction in the number of axons per bundle as well as defects in larval crawling behavior. These results were interpreted as evidence that wrapping glia are required to promote the survival of larval abdominal nerves.
This is likely true, however, it is possible that the Gal4 has non-specific expression at earlier developmental stages and since its activity has not been restricted in time with a Gal80ts, strictly speaking the authors have not ruled out that the reduction in neuronal numbers could be caused by a developmental defect rather than a later survival phenotype.
Perhaps the authors can restrict Gal4 expression during development or temper their conclusion in the text accordingly.
2. The authors perform an RNAi screen in wrapping glia to identify how cell-surface and secreted proteins might play a role and identify Ddr as a potential candidate. They saw a significant decrease in the wrapping index when Ddr was knocked down by RNAi or in LOF mutants they generated. The authors conclude that Ddr is required for normal wrapping glia morphogenesis. It is important that the authors quantify glial numbers in controls or when Ddr is knocked down or in Ddr mutants to make the argument that the Ddr LOF phenotype is due to a morphogenesis defect rather than a glial survival defect.
3. The authors then sought to determine how Ddr is activated and focus on Multiplexin (Mp), a collagen and predicted ligand of Ddr that was also a hit from their screen. Ddr and Mp showed a genetic interaction.
a. The authors should provide quantifications of the wrapping index for Mp RNAi in wrapping glia.
b. The Mp-GFP protein trap expression is not suitable to identify exactly which cells are producing Mp- i.e. glia, neurons or both. It would be good to clarify this: is there an enhancer trap or does knocking down Mp in wrapping glia alone abolish GFP expression (Mp RNAi or degrad GFP or GFP RNAi)?
4. It would be helpful if the authors attempted to place Ddr in context.
While they have added a new component, our understanding of how glial wrapping is controlled has not been advanced much. For example integrins have been implicated in glial wrapping and are known to interact with ECM components including collagens. Do Mys and Mp interact genetically? Do Mys and Ddr interact genetically? Could they be functioning in the same pathway? Is it known whether other factors (like integrins, htl, etc.) affect axon caliber?
5. Finally, although there is a clear ensheathment defect when Ddr is knocked down, there was no effect on larval crawling behavior. Were there some hits from the screen that did cause behavioral defects while others

© 2022. Published by The Company of Biologists under the terms of the Creative Commons Attribution License (https://creativecommons.org/licenses/by/4.0/).

(like Ddr) did not? What about KD of htl or vn, etc.? Can these shed light on or decouple the likely multiple roles that glia are performing in this context?
a. If only genetic ablation causes behavioral deficits, then the authors should temper the text significantly.
In particular, the introduction emphasizes that wrapping is critical to neuronal function, but this may simply not be the case in Drosophila. It does not necessarily detract from the goal of identifying regulators of wrapping.

Minor points:
6. In Fig. 1 the authors show that nrv2-Gal4 is not specific to wrapping glia and create a split-Gal4 for wrapping glia-specific expression, however all later experiments use either nrv2-Gal4 or Repo-Gal4. Given that we now know that nrv2-Gal4 expression is non-specific, the authors should provide some experiments to confirm their Ddr and Mp knockdowns using the wrapping glia split-gal4 line.
7. It would be good to show that Ddr is expressed in the wrapping glia.
8. To support the argument that Mp is acting in an autocrine manner, the authors should also knockdown Mp in neurons to show that glial wrapping is unaffected.

<u>Reviewer 3</u>

Advance summary and potential significance to field

The close association of axons with glial branches is essential for their functioning. In vertebrates, several glial cell classes ensheath axons by myelination. However, important glial cell types, such as ensheathing glia in the olfactory nerve enwrap individual axons or axon bundles but do not form myelin. This is a feature shared with invertebrate glia. However both in vertebrates and insects the molecular mechanisms controlling ensheathment of axons by non-myelinating glia remains largely unknown. In this study, Megan Corty, Marc Freeman and colleagues identified an distinct role for the Discoidin domain receptor, a tyrosine kinase, which in connection with the collagen Multiplexin controls axon ensheathment by wrapping glia in the larval peripheral nerves of Drosophila. A comprehensive, analysis combining genetic imaging (with beautiful EM images) and behavioral approaches (crawling behavior), elucidates the underlying mechanisms and implications for nervous system function. Moreover, the study provides an interesting comparison of the role of Ddr in larval peripheral and adult L1 wing nerves, where Ddr is rather involved in neuron survival and axon caliber regulation. The manuscript is very well written, the conclusions are supported by high quality images and quantifications, and are carefully interpreted.
Finally, the notion that Ddr and Collagen have a conserved role in glial ensheathment in vertebrates and invertebrates further adds to the interest of the study.

Comments for the author

Suggestions and comments to strengthen the manuscript
1. The study reveals a novel function of Ddr and the collagen Multiplexin in controlling ensheathment of axons by wrapping glia using comprehensive genetic tests combined with high resolution EM analysis.
However, one aspect which is potentially missing is the localization/expression of the proteins to provide deeper insights into their role in promoting branching of processes?
2. Another question is whether they are purely required in glia or by both neurons and glia?
3. In Figure 1, it would be helpful to indicate the nuclei in all channels, to more easily assign the nuclei to specific nerves and to assess the numbers of cell bodies/nerve.
4. The authors describe that several nerves showed abnormal hypertrophy of the outer perineurial glia layer, following the killing of wrapping glia. It would be helpful to provide a n number to convey whether this is rare or more common event.
5. Oaz in line 89 would need to be defined. Similarly in line 100, the FIMTracker would need to be introduced. In line 122, the term “tight honeycomb” is perhaps not ideal as it evokes some hexagonal outlines, or it is just difficult to see in the provided image?
6. In line 145, it would help to describe the origin of the mutant alleles and how they were generated (it is in the figure legend, but should be mentioned in the main text). In line 155, it would be important to specify the origin and nature of the BAC.

© 2022. Published by The Company of Biologists under the terms of the Creative Commons Attribution License (https://creativecommons.org/licenses/by/4.0/).

7. Page 9. The ensheathment phenotype is very clear, however, the nerves seemed to be smaller in larvae lacking Ddr, especially in the transheterozygous mutant. Is this meaningful?
8. Figure 4. Ensheathing phenotypes relative to crawling behavior on average does not show significant differences in the mutant compared to the controls. However, the phenotypes are variable, and thus the question arises whether the existing data could allow a correlation – reduced crawling correlates with reduced ensheathment (i.e. larvae with behavioral defects were then processed for EM?)
9. For Figure 5, it would be helpful to show also some labeled wings to better understand the point that there a fewer healthy cell bodies, as it is not clear how they were judged to be healthy or unhealthy.

First revision

Author response to reviewers' comments

We appreciate your suggestions and feel they have strengthened the study. Please see our responses to each comment below, in blue. In the annotated revised manuscript major text changes made in response to these suggestions are similarly colored in blue.
(We have uploaded the fully formatted version with colored text as a Supplementary file.)

Reviewer 1 Advance Summary and Potential Significance to Field:
"Discoidin domain receptor regulates ensheathment, survival, and caliber of peripheral axons" by Corty et al reports a set of experiments employing Drosophila as a model, which have uncovered several novel roles for both wrapping glia and the Discoidin domain receptor in regulating peripheral nerve formation and maintenance. The first set of data in the manuscript focus on the analysis of the consequences of specifically ablating peripheral wrapping glia (through a clever gene targeting approach), finding that this disrupts both axonal integrity and larval fly behaviour. From there, the manuscript describes a series of studies that aimed to better understand the mechanisms of nerve wrapping, particularly given that the multi-axon wrapping seen in larval Drosophila nerves, analogous to that which is mediated by Remak Schwann cells in vertebrates, is relatively understudied. A powerful wrapping glia-driven RNAi-based screen identified several factors that disrupted the morphology of wrapping glia, of which Ddr was one, and the principal focus on this manuscript. The identificaiton of ddr as a regulator of wrapping glia morphology was corroborated by the generation of mutant lines through CRISPR-mediated gene targeting. In addition to characterising phenotypes in both constitutive and wrapping glia specific ddr mutants in the larva and adult fly, the study also identifies a Drosophila Collagen as a likely ligand for ddr in mediating wrapping glial cell morphology in the larval stage. There is an abundance of important and novel information in this manuscript and the conclusions drawn are generally very well supported. Future studies will determine whether the function of ddr will be conserved in the vertebrate nervous system.

Reviewer 1 Comments for the Author:
While some may consider testing conservation of ddr function in a vertebrate an important question to address experimentally up front, I personally think that the study is of a sufficient enough advance to warrant publication as a standalone Drosophila-focused piece and for vertebrate conservation to be tackled separately. This said, I do, however, have a number of points that I think need to be addressed before publication, some of which are suggestions for extending the analyses a bit further, and some of which will be requests for clarification based on my lack of direct expertise in fly genetics.

1. Regarding wrapping glia ablation, can the authors please measure axon diameter/ caliber in animals without wrapping glia, given that the TEM data is to hand. This would help better match up and understand the similarities and differences in phenotypes between larvae and adults (discussed further below).

Thank you for this suggestion. We have performed this analysis and found that the median axon size is smaller in ablated vs control nerves. Unlike analysis of the dTSM axon where we can directly compare size of a known axon between conditions, these are population assessments. One

© 2022. Published by The Company of Biologists under the terms of the Creative Commons Attribution License (https://creativecommons.org/licenses/by/4.0/).

potential caveat with the ablation data is that we saw fewer axon profiles in the ablated nerves—so is the shift in sizes due to loss of a specific population or rather smaller sizes among remaining axons? To account for this, we performed this analysis only using nerves that had at least 70 axon profiles, and we still found a similar difference in the median size suggesting it is an effect on axon size and not just survival. We report this finding in the text and Sup Fig S12. We also performed this caliber analysis in our *Ddr* larval nerves and found that median axon size is similarly reduced in *Ddr* mutant vs control nerves, also reported in the text and Fig. S12. Thus Ddr has similar effects in promoting increased caliber at both stages.

2. Related to this point, is it possible to ablate wrapping glia in the adult, using the new split gal4 model, and if so, can the effect on diameter be assessed. I wouldn't consider this an essential request, but rather a potential extension, depending on feasibility.

Thank you for this suggestion. Animals with ablated wrapping glia can survive to adulthood. We have analyzed these animals for effects on neuronal survival by fluorescence, and that data is already included in another manuscript focusing on long term glia support of axons in the wing. We do not have TEM data for adult dTSM axon diameter analysis at this time.

3. I am unclear, not being a fly expert, whether the wrapping glia of larvae are the same cells in the adult. It appears that the mechanisms of wrapping are quite distinct, with multi-axon wrapping prominent in larvae, and single axon wrapping prominent in the adult. Do the same cells move to wrapping fewer axons, how does cell number increase over time, or is the population turned over. To me, the most unclear aspect of the study was the disconnect between larval and adult phenotypes. I think that non-experts would benefit greatly from knowing how wrapping glia of larvae and adults were related. Similarly, I think it would be important to actually quantify wrapping glial cell number in larvae and adults. Is the number reduced in larvae, or simply the morphology? If also number, does this catch up in adult, explaining lack of obvious wrapping phenotype.

Thank you for pointing this out as we are sure that many readers will have the same questions, and we should have included this information, some of which was inadvertently trimmed during editing for length. We now have an explanation for this in the text. To summarize, the wrapping glia cells that ensheathe larval nerves are distinct from those in the wing. Larval abdominal nerve wrapping glia are born in the embryo. There are 3 WG cells to cover the nerve, each covering a stereotyped territory along the nerve. Interestingly, it is just a single wrapping glia cell that covers the Nerve Elongation Region (NER), i.e., the nerve from where it exits the VNC to where it attaches to the body wall in the periphery. Thus, in the larvae, a single WG cell is “responsible” for ensheathing all ~80 axons where we perform our analysis. Hence, we did not initially quantify this parameter. We have now double checked this and include these results in the text that there is no change in wrapping glia cell number in the mutant larvae. The glia in the wing arise from within the wing imaginal disc and so are newly born with that genesis of that nerve. There are ~40 wrapping glia along the wing nerve, with ~13 covering the region where we analyze wrapping. Given their spacing we estimate that when analyzing wing nerve cross sections, we may be seeing processes from 2-4 wrapping glia cells. We do agree that glial number might be important here, so we have also quantified wrapping glia cell number in the wing in 4-5dpe *Ddr* mutant and control animals (the time point we analyzed ensheathment) and found no significant difference in the number of wrapping glia between these groups, now reported in the text and a Supplemental Figure. We do think that the increased individual ensheathment in the adult may, in part, be due to increased wrapping capacity of multiple vs one cell over a shortened distance. We include a brief discussion of this.

4. Again, related to larval to adult analyses, I'm afraid I wasn't able to tell if the cell-type specific driver (repo) used to disrupt ddr in adults were such that larval ddr function was intact and ddr was only disrupted in adult wrapping glia. This could simply be stated for clarity and would add strength to interpretation of adult-specific phenotypes (or lack thereof). One point that is not clear in this regard is to what extent phenotypes have transitioned from being wrapping- glia specific to broader glial specific. Are any of the adult phenotypes truly wrapping glia specific? Much as per the question regarding adult wrapping glia ablation, I don't know whether it is possible to do wrapping glia specific targeting of ddr through to adult stages. I just fear that naive readers might conflate

© 2022. Published by The Company of Biologists under the terms of the Creative Commons Attribution License (https://creativecommons.org/licenses/by/4.0/).

wrapping glia functions in larvae with glial functions in adults. It would be important to pre-empt this confusion/ conflation.

Thank you for pointing out these instances where things were not clear. In the adult experiments using *repo-Gal4*, Ddr would have been knocked down throughout the animal's life (in all glia), and then analyzed in adults. This was originally chosen to maximize the strength of the knockdown (Repo is a strong driver) as well as chromosomal location. In response to your question about WG-specificity (and suggestions from other reviewers), we have now repeated the adult neuronal survival assay using the *WGSplitGal4* driver to knock down Ddr specifically in wrapping glia and found that we still saw a reduction in neuronal survival at 28 days post eclosion (with no initial difference at 4 dpe). We have added these data but also left in the repo experiments since they correspond to our extensive TEM quantifications. We have also checked our language and clarified text in several places to address these concerns.

Minor point for discussion. There is a disconnect in the behavioural consequences of wrapping glia ablation and ddr loss of function, which is discussed. However, could it also be that the effect of the wrapping glia ablation on behaviour is due to the ablation aspect, i.e. the induciton of cell death and associated inflammatory response? This could be discussed, as it is broadly relevant to studies that ablate cells and assess behaviour: how do such manipulations reflect essential function of the cell type versus the inevitable parallel consequences of cell death.

We now include this potential caveat in our discussion of these results.

Reviewer 2 Advance Summary and Potential Significance to Field:
In this study, Corty et al. set out to dissect the molecular mechanisms that govern wrapping glial ensheathment of peripheral nerves, a process which is incompletely understood. Focusing on the larval abdominal neurons, the authors used an RNAi screen against cell-surface and secreted proteins in wrapping glia and identified a new molecular player: the Discoidin Domain Receptor (Ddr) as a regulator of glial ensheathment in larval nerves. Overall, while the manuscript is well-written and clear, the authors could do more to better place the role of Ddr in context of other known glial wrapping regulators and advance our understanding of the process beyond merely adding a new player. In addition, several points need to be addressed to support some of the interpretations of the manuscript.

Reviewer 2 Comments for the Author:
1. To demonstrate that wrapping glia are essential to support neural circuit function, the authors genetically ablated wrapping glia. As a result, they observed a significant reduction in the number of axons per bundle as well as defects in larval crawling behavior. These results were interpreted as evidence that wrapping glia are required to promote the survival of larval abdominal nerves. This is likely true, however, it is possible that the Gal4 has non-specific expression at earlier developmental stages and since its activity has not been restricted in time with a Gal80ts, strictly speaking the authors have not ruled out that the reduction in neuronal numbers could be caused by a developmental defect rather than a later survival phenotype. Perhaps the authors can restrict Gal4 expression during development or temper their conclusion in the text accordingly.

Thank you for this suggestion. We agree that we cannot completely rule out this possibility, and have tried to address this caveat in the text. Unfortunately, the Split-Gal4 was built with a VP16 activation domain (rather than Gal4 activation domain) that makes it insensitive to Gal80 repression, so we cannot perform the exact experiment suggested.

2. The authors perform an RNAi screen in wrapping glia to identify how cell-surface and secreted proteins might play a role and identify Ddr as a potential candidate. They saw a significant decrease in the wrapping index when Ddr was knocked down by RNAi or in LOF mutants they generated. The authors conclude that Ddr is required for normal wrapping glia morphogenesis. It is important that the authors quantify glial numbers in controls or when Ddr is knocked down or in Ddr mutants to make the argument that the Ddr LOF phenotype is due to a morphogenesis defect rather than a glial survival defect.

In addition to this and other reviewer comments we realized that we accidentally removed information explaining that the area we analyze in larval nerves actually only has a single wrapping

© 2022. Published by The Company of Biologists under the terms of the Creative Commons Attribution License (https://creativecommons.org/licenses/by/4.0/).

glia cell. In addition to this text clarification, we also performed a quantification of nuclei number in control and Ddr mutant larval and adult nerves and found that it is unchanged. In the wing, there are many more wrapping glia, so this is an important control. We have also now quantified this and found no difference in wrapping glia cell number between control and Ddr mutants. These new data are all included in the revised manuscript. Taken together we think these results support our conclusions that this is a morphological defect rather than a glial survival defect.

3. The authors then sought to determine how Ddr is activated and focus on Multiplexin (Mp), a collagen and predicted ligand of Ddr that was also a hit from their screen. Ddr and Mp showed a genetic interaction.
a. The authors should provide quantifications of the wrapping index for Mp RNAi in wrapping glia.

We have included quantification for the wrapping phenotype for nrv2>MpRNAi and this is now included in Figure 7.

b. The Mp-GFP protein trap expression is not suitable to identify exactly which cells are producing Mp- i.e. glia, neurons or both. It would be good to clarify this: is there an enhancer trap or does knocking down Mp in wrapping glia alone abolish GFP expression (Mp RNAi or degrad GFP or GFP RNAi)?

Given that Mp is a secreted protein, we agree that the Mp-GFP trap is not adequate to pinpoint the source of Mp, and were mainly presenting it to show Mp was present in the peripheral nerves. Our best evidence that the *relevant* source of Mp for wrapping glial morphogenesis is wrapping glia themselves is genetic evidence from our findings that
1) KD of Mp in wrapping glia is sufficient to cause a phenotype and 2) KD of Mp in neurons (a new experiment in the revised manuscript) does not cause a phenotype. That said, we cannot completely rule out that neurons do express Mp, only that knocking it down in those cells does not affect the process we are studying. Moreover, there are reports in the literature that other peripheral glia cells can express Mp based on the same Mp-GFP pattern. We cannot formally rule out contribution of Mp from these other glia, although KD in WG alone is sufficient for the phenotype arguing that contribution of WG-derived Mp is significant for ensheathment.

In response to your request for an experiment using RNAi against Mp in the Mp-GFP background, we performed this (with the nrv2 driver) and found it did not completely eliminate GFP-puncta from the nerve, suggesting that multiple cell types may be expressing Mp (or that KD is incomplete). We have adjusted the text to more clearly reflect these caveats, but do continue to emphasize that our WG-specific knockdown experiments indicate that wrapping glia are an important source of Mp in driving their morphogenesis.

4. It would be helpful if the authors attempted to place Ddr in context. While they have added a new component, our understanding of how glial wrapping is controlled has not been advanced much. For example, integrins have been implicated in glial wrapping and are known to interact with ECM components including collagens. Do Mys and Mp interact genetically? Do Mys and Ddr interact genetically? Could they be functioning in the same pathway? Is it known whether other factors (like integrins, htl, etc.) affect axon caliber?

Thank you for this suggestion. It does remain unclear how Ddr signaling interacts with other known regulators of glial wrapping. We attempted transheterozygous experiments with Mys and Ddr (as with Mp and Ddr) and did not find a wrapping phenotype. However, dominant genetic interactions are somewhat rare, so we cannot completely rule out the possibility that Ddr and integrin signaling pathways interact. We have expanded the discussion to include speculation how Ddr might be acting with other known pathways including Mys. These are also very important experiments for the future.

5. Finally, although there is a clear ensheathment defect when Ddr is knocked down, there was no effect on larval crawling behavior. Were there some hits from the screen that did cause behavioral defects while others (like Ddr) did not? What about KD of htl or vn, etc.? Can these shed light on or decouple the likely multiple roles that glia are performing in this context?

© 2022. Published by The Company of Biologists under the terms of the Creative Commons Attribution License (https://creativecommons.org/licenses/by/4.0/).

This discrepancy was also surprising to us, however as we note in the text, the Klambt lab has reported similar findings when disrupting *htl* signaling, which also strongly impairs ensheathment, with only very minor behavioral defects compared to ablations, which disrupted crawling similar to our results. We do agree this implies that ensheathment *per se* is not strictly required for neurons to function mostly normally—at least in the larvae. We note (in the text) that because wrapping is progressive even wild type 1st and 2nd instar larvae can and do have coordinated behavior (hatching, crawling) with minimal to no ensheathment, and even in wild type 3rd instars many axons are not individually wrapped. The severe behavioral defects could be specifically due to the seemingly "missing" axons observed in TEM—are whole neurons missing due to a developmental or survival defect? Have their axons been misrouted? We do not see this loss of profiles in Ddr mutants, so this might also explain the discrepancy. And as another reviewer suggests, there might be "collateral damage" from the act of ablation itself. We try to address each of these possibilities as requested in the discussion.

a. If only genetic ablation causes behavioral deficits, then the authors should temper the text significantly.
In particular, the introduction emphasizes that wrapping is critical to neuronal function, but this may simply not be the case in Drosophila. It does not necessarily detract from the goal of identifying regulators of wrapping.

We have altered the text as suggested, including noting that even vertebrate ensheathing glia have functions that are separable from wrapping. We agree that these data suggest that wrapping *per se* is not required (at least at larval stages) for neuronal function, at least in the simple behavioral assays that we have performed.

Minor points:
6. In Fig. 1 the authors show that nrv2-Gal4 is not specific to wrapping glia and create a split-Gal4 for wrapping glia- specific expression, however all later experiments use either nrv2-Gal4 or Repo-Gal4. Given that we now know that nrv2-Gal4 expression is non-specific, the authors should provide some experiments to confirm their Ddr and Mp knockdowns using the wrapping glia split-gal4 line.

Thank you for this feedback. We have tried to clarify in the text that nrv2-Gal4 is still very useful for manipulating wrapping glia. It has been the standard in the field for manipulating wrapping glia for morphogenesis studies for years because it is only drives in wrapping glia in the nerves—no neuronal expression, no SPG or PG expression. It is also a strong, consistent driver. The SplitGal4 was necessary in order to perform ablation and or behavioral experiments, as having even low level KD of genes in astrocytes in the CNS (or killing them) could affect behavior independently. (And in spite of the order of the text, the screen and many experiments were completed using nrv2 before the SplitGal4 was generated.) That said, we have now performed both larval morphogenesis and adult neuron survival experiments using WrappingGlia-SplitGal4 and show that we can replicate the key nrv2 and repo findings. These new data are included in the revised manuscript.

7. It would be good to show that Ddr is expressed in the wrapping glia.

We agree and have tried to make an antibody to Ddr—the gold standard to show expression—without success. That said, we believe that the WG-specific knockdown provided by the nrv2-gal4 and WGSplitGal4 experiments, combined with the cell type specific rescue experiments do provide strong genetic evidence that Ddr is required in (and therefore expressed in) wrapping glia.

8. To support the argument that Mp is acting in an autocrine manner, the authors should also knockdown Mp in neurons to show that glial wrapping is unaffected.

We have performed this experiment using the pan-neuronal driver elav-Gal4 and found that there is no effect on wrapping glia morphology. These data are now included in Figure 7.

Reviewer 3 Advance Summary and Potential Significance to Field:
The close association of axons with glial branches is essential for their functioning. In vertebrates, several glial cell classes ensheath axons by myelination. However, important glial cell types, such as ensheathing glia in the olfactory nerve enwrap individual axons or axon bundles but do not form myelin. This is a feature shared with invertebrate glia. However both in vertebrates and insects the

© 2022. Published by The Company of Biologists under the terms of the Creative Commons Attribution License (https://creativecommons.org/licenses/by/4.0/).

molecular mechanisms controlling ensheathment of axons by non-myelinating glia remains largely unknown. In this study, Megan Corty, Marc Freeman and colleagues identified an distinct role for the Discoidin domain receptor, a tyrosine kinase, which in connection with the collagen Multiplexin controls axon ensheathment by wrapping glia in the larval peripheral nerves of Drosophila. A comprehensive, analysis combining genetic, imaging (with beautiful EM images) and behavioral approaches (crawling behavior), elucidates the underlying mechanisms and implications for nervous system function. Moreover, the study provides an interesting comparison of the role of Ddr in larval peripheral and adult L1 wing nerves, where Ddr is rather involved in neuron survival and axon caliber regulation. The manuscript is very well written, the conclusions are supported by high quality images and quantifications, and are carefully interpreted.
Finally, the notion that Ddr and Collagen have a conserved role in glial ensheathment in vertebrates and invertebrates further adds to the interest of the study.

Reviewer 3 Comments for the Author:
Suggestions and comments to strengthen the manuscript

1. The study reveals a novel function of Ddr and the collagen Multiplexin in controlling ensheathment of axons by wrapping glia using comprehensive genetic tests combined with high resolution EM analysis. However, one aspect which is potentially missing is the localization/expression of the proteins to provide deeper insights into their role in promoting branching of processes?

Thank you for this suggestion. While we agree that this would be very useful information, our attempts to make a Ddr antibody have not yet succeeded. It also seems like more than just showing expression in a cell type, getting subcellular resolution (i.e. axon-facing vs glia-facing membranes) would be ideal, which may be achievable via immuno EM, but we have not been able to apply this technique to this problem. We do feel that we have strong genetic evidence (made stronger via your suggested experiments) that Ddr is required in wrapping glia and Mp is at least predominantly supplied by wrapping glia (see above discussion.)

2. Another question is whether they are purely required in glia or by both neurons and glia? For Ddr: For Mp:

We have now performed KD experiments with elav-Gal4 and found that KD of neither Ddr nor Mp in neurons affects wrapping glia morphology. This new data is included in the revised manuscript.

3. In Figure 1, it would be helpful to indicate the nuclei in all channels, to more easily assign the nuclei to specific nerves and to assess the numbers of cell bodies/nerve.

Thank you for this great suggestion. We have altered this figure as requested and agree it is much easier to interpret the figure.

4. The authors describe that several nerves showed abnormal hypertrophy of the outer perineurial glia layer, following the killing of wrapping glia. It would be helpful to provide a n number to convey whether this is rare or more common event.

Thank you for this suggestion. We reported the % but only in the Supplemental Legend. We have now moved this to the main text.

5. Oaz in line 89 would need to be defined. Similarly in line 100, the FIMTracker would need to be introduced. In line 122, the term “tight honeycomb” is perhaps not ideal as it evokes some hexagonal outlines, or it is just difficult to see in the provided image?

Thank you for pointing this out. Several of these descriptions were lost when editing to reduce word count. For Oaz and the FIMTracker we have added additional descriptions in the main text and also pointed more clearly to the Methods section where more detail can be found.

In regards to the “honeycomb” term, we had taken to using this phrasing internally in lab but you are correct it is not a strict hexagonal feature so we have changed the wording to avoid confusion.

© 2022. Published by The Company of Biologists under the terms of the Creative Commons Attribution License (https://creativecommons.org/licenses/by/4.0/).

6. In line 145, it would help to describe the origin of the mutant alleles and how they were generated (it is in the figure legend, but should be mentioned in the main text). In line 155, it would be important to specify the origin and nature of the BAC.

Thank you for this, again some detail was cut out during editing for length but we have added a brief description to the main text in addition to more details in the methods, which we also more clearly direct the readers to.

7. Page 9. The ensheathment phenotype is very clear, however, the nerves seemed to be smaller in larvae lacking Ddr, especially in the transheterozygous mutant. Is this meaningful?

There is considerable variability in nerve size across nerves across and between animals. This is in part due to the sporadic presence of a glial nucleus in a given cross section. Across the nerves we've analyzed this does not score as a significant difference between genotypes.

8. Figure 4. Ensheathing phenotypes relative to crawling behavior on average does not show significant differences in the mutant compared to the controls. However, the phenotypes are variable, and thus the question arises whether the existing data could allow a correlation – reduced crawling correlates with reduced ensheathment (i.e. larvae with behavioral defects were then processed for EM?)

Thank you for these suggestions. As noted above similar (lack of) behavioral phenotypes were seen in FGFR mutants. Although we did retain some of the experimental animals from crawling experiments to double check the genotype/phenotypes, we did not keep track of specific animals that would correspond to specific crawling traces so cannot provide the specific breakdown requested. That said we do have plans in the future to test a variety of other screen hits showing varying degrees of impaired ensheathment to see if a correlation between severity and behavior can be ascertained. We will keep in mind to perform some individual animal traces with subsequent TEM processing of individual larvae to see if within animal differences also may contribute.

9. For Figure 5, it would be helpful to show also some labeled wings to better understand the point that there a fewer healthy cell bodies, as it is not clear how they were judged to be healthy or unhealthy.

Thank you for pointing out this omission. We now include examples both healthy and unhealthy cell bodies (and often there are simply fewer cell bodies) in Supplemental Figure S10.

Second decision letter

MS ID#: DEVELOP/2022/200636

MS TITLE: Discoidin domain receptor regulates ensheathment, survival, and caliber of peripheral axons

AUTHORS: Megan M Corty, Alexandria L Hulegaard, Jo Q. Hill, Amy E Sheehan, Sue A Aicher, and Marc R Freeman
ARTICLE TYPE: Research Article

I am happy to tell you that your manuscript has been accepted for publication in Development, pending our standard ethics checks.

Reviewer 1

Advance summary and potential significance to field

The authors have addressed my points through both further experimentation, analysis, careful clarification and discusison, and in my opinion this manuscript is suitable for publication and will make a very nice addition to the field.

© 2022. Published by The Company of Biologists under the terms of the Creative Commons Attribution License (https://creativecommons.org/licenses/by/4.0/).

Comments for the author

No further comments

Reviewer 2

Advance summary and potential significance to field

Corty et al. identify a new molecular player in glial wrapping of neuronal axons:
the Discoidin Domain Receptor (Ddr). They found that Ddr LOF in wrapping glia resulted in poor axon ensheathment and reduced axon calibre, and provide data in support of a model where Ddr is activated by Mp, also produced by wrapping glia.
The data presented in this study begin to dissect out how wrapping glia contribute to nerve development, maintenance, and function through axon ensheathment and by other mechanisms, yet to be identified.

Comments for the author

The authors have provided additional experiments to address where Ddr and Mp are expressed. They also provided quantifications requested in revisions, text clarifications and amendments to the discussion. Finally, the authors have attempted to place Ddr in the context of other known regulators of glial wrapping.
Although the results of these experiments were inconclusive, I am satisfied with revisions and believe that the manuscript is suitable for publication.

Reviewer 3

Advance summary and potential significance to field

In this study, Megan Corty, Marc Freeman and colleagues identified a novel role for the Discoidin domain receptor Ddr in controlling axonal ensheathment in developing peripheral nerves by wrapping glia in Drosophila. This molecular mechanism in the still poorly understood process of axonal ensheathment has the potential to be conserved among vertebrates and invertebrates and thus will be of wide interest in the field of glial biology.

Comments for the author

The authors carried out very careful revisions, including new additional experiments (e.g. neuronal KDs), which greatly strengthened the conclusions of the study. The authors addressed all my concerns where it was technically possible (expression patterns remain difficult to assess for some determinants, and thus genetic tests are acceptable as replacements). In summary, I look forward to seeing this interesting and beautiful study in print.

© 2022. Published by The Company of Biologists under the terms of the Creative Commons Attribution License (https://creativecommons.org/licenses/by/4.0/).
